# Computational evaluation of psoas muscle influence on walking function following internal hemipelvectomy with reconstruction

**DOI:** 10.3389/fbioe.2022.855870

**Published:** 2022-09-28

**Authors:** Marleny M. Vega, Geng Li, Mohammad S. Shourijeh, Di Ao, Robert C. Weinschenk, Carolynn Patten, Josep M. Font-Llagunes, Valerae O. Lewis, Benjamin J. Fregly

**Affiliations:** ^1^ Rice Computational Neuromechanics Lab, Department of Mechanical Engineering, Rice University, Houston, TX, United States; ^2^ Department of Orthopaedic Surgery, University of Texas Southwestern Medical Center, Dallas, TX, United States; ^3^ Biomechanics, Rehabilitation, and Integrative Neuroscience (BRaIN) Lab, UC Davis School of Medicine, Sacramento, CA, United States; ^4^ UC Davis Center for Neuroengineering and Medicine, University of California, Davis, CA, United States; ^5^ VA Northern California Health Care System, Martinez, CA, United States; ^6^ Biomechanical Engineering Lab, Department of Mechanical Engineering and Research Centre for Biomedical Engineering, Universitat Politècnica de Catalunya, Barcelona, Spain; ^7^ Health Technologies and Innovation, Institut de Recerca Sant Joan de Déu, Esplugues de Llobregat, Spain; ^8^ Department of Orthopaedic Oncology, University of Texas MD Anderson Cancer Center, Houston, TX, United States

**Keywords:** orthopedic biomechanics, neuromusculoskeletal modeling, computational modeling, predictive simulation, treatment optimization, optimal control, pelvic sarcoma, internal hemipelvectomy surgery

## Abstract

An emerging option for internal hemipelvectomy surgery is custom prosthesis reconstruction. This option typically recapitulates the resected pelvic bony anatomy with the goal of maximizing post-surgery walking function while minimizing recovery time. However, the current custom prosthesis design process does not account for the patient’s post-surgery prosthesis and bone loading patterns, nor can it predict how different surgical or rehabilitation decisions (e.g., retention or removal of the psoas muscle, strengthening the psoas) will affect prosthesis durability and post-surgery walking function. These factors may contribute to the high observed failure rate for custom pelvic prostheses, discouraging orthopedic oncologists from pursuing this valuable treatment option. One possibility for addressing this problem is to simulate the complex interaction between surgical and rehabilitation decisions, post-surgery walking function, and custom pelvic prosthesis design using patient-specific neuromusculoskeletal models. As a first step toward developing this capability, this study used a personalized neuromusculoskeletal model and direct collocation optimal control to predict the impact of ipsilateral psoas muscle strength on walking function following internal hemipelvectomy with custom prosthesis reconstruction. The influence of the psoas muscle was targeted since retention of this important muscle can be surgically demanding for certain tumors, requiring additional time in the operating room. The post-surgery walking predictions emulated the most common surgical scenario encountered at MD Anderson Cancer Center in Houston. Simulated post-surgery psoas strengths included 0% (removed), 50% (weakened), 100% (maintained), and 150% (strengthened) of the pre-surgery value. However, only the 100% and 150% cases successfully converged to a complete gait cycle. When post-surgery psoas strength was maintained, clinical gait features were predicted, including increased stance width, decreased stride length, and increased lumbar bending towards the operated side. Furthermore, when post-surgery psoas strength was increased, stance width and stride length returned to pre-surgery values. These results suggest that retention and strengthening of the psoas muscle on the operated side may be important for maximizing post-surgery walking function. If future studies can validate this computational approach using post-surgery experimental walking data, the approach may eventually influence surgical, rehabilitation, and custom prosthesis design decisions to meet the unique clinical needs of pelvic sarcoma patients.

## Introduction

**FIGURE 1:**
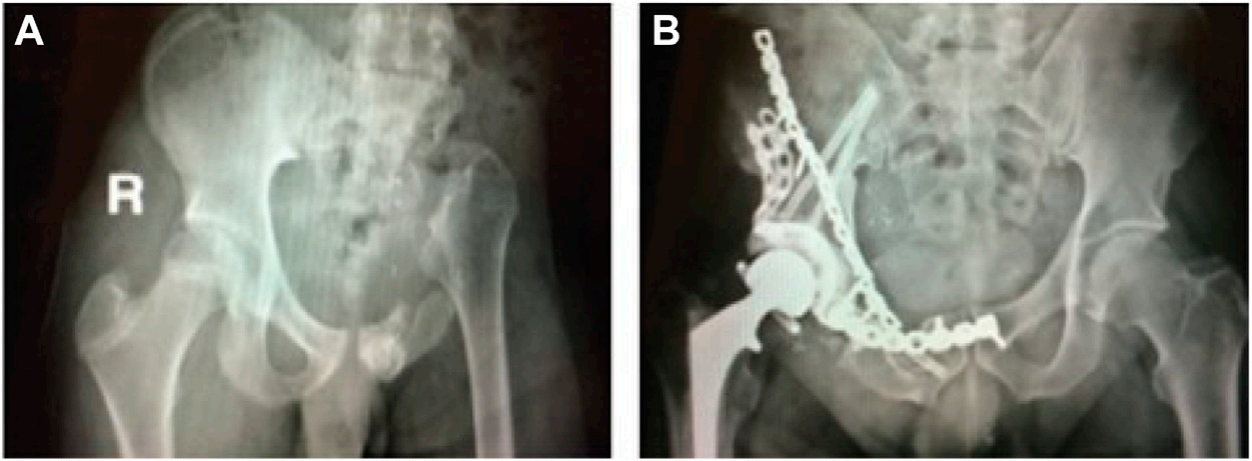
**(A)** Sample x-ray image of a pelvic cancer patient who received internal hemipelvectomy surgery with no reconstruction. **(B)** Sample x-ray image of a pelvic cancer patient who received internal hemipelvectomy surgery with allograph reconstruction and a total hip replacement. Images courtesy of Dr. Valerae Lewis, MD Anderson Cancer Center patient education DVD.

Over the past few decades, surgical treatment of pelvic sarcomas has improved considerably with limb-preserving internal hemipelvectomy frequently being possible in place of limb-sacrificing external hemipelvectomy to achieve local control of cancerous tumors (Aljassir et al., 2005). When limb salvage is possible, the orthopedic oncologist must choose between three surgical options: 1) no reconstruction, 2) allograft reconstruction, or more recently, 3) custom prosthesis reconstruction (Figure 1). For all three options, when the hip joint and numerous muscles are removed to achieve clear margins around the tumor, restoration of normal ambulatory function can be challenging. Even when the hip joint must be sacrificed, patients who receive no reconstruction typically ambulate well post-surgery and benefit from a reduced complication rate compared to the other two options (Gupta et al., 2020). However, the recovery time is on the order of 6 months, and the resulting leg-length discrepancy is uncomfortable, requires a shoe lift, and often results in scoliosis due to altered gait mechanics. Patients who receive allograft reconstruction with total hip replacement typically achieve a post-surgery ambulatory function that is closer to normal since leg shortening and medialization are avoided. However, the recovery time is on the order of a year or more, and there is a high risk of complications and additional surgery due to the relatively short lifespan of the alloprosthesis (Beadel et al., 2005). The goals of custom prosthesis reconstruction with a total hip replacement are to maximize post-surgery ambulatory function and minimize recovery time. Though custom prosthesis reconstruction has the best clinical potential, the design of durable custom prostheses remains challenging, with a three-year survival rate of less than 70% (Sun et al., 2011).

Based on the potential benefits of custom prosthesis reconstruction, orthopedic implant companies and academic researchers have started to design and implant custom pelvic prostheses that include a total hip replacement (Hilton et al., 2017; Dion et al., 2018; Bai et al., 2019; Babazadeh Naseri et al., 2021). Custom pelvic prostheses are designed using patient-specific three-dimensional pelvis geometric models developed from a CT scan of the patient’s pelvis (Chen et al., 2020). Since the shape of the custom prosthesis normally recapitulates the removed bony anatomy, an implicit design assumption is that mimicking the original anatomy will produce the best post-surgery ambulatory function. However, anatomic reconstruction may not produce the best post-surgery function in other areas of the body when numerous muscles are injured or removed. For example, a reverse shoulder prosthesis switches the ball and shallow socket between the humerus and scapula to improve mechanical stability by accounting for missing or impaired rotator cuff muscles (Gutiérrez et al., 2007). Thus, it is possible that a non-anatomic custom pelvic prosthesis design could achieve better functional outcomes than current anatomic designs. Regardless, the current custom prosthesis design process does not account for the post-surgery loading patterns that the prosthesis and remaining bone will experience, which could help explain why prosthesis durability is not as high as desired. Furthermore, the current design process provides no means for predicting how different surgical or rehabilitation decisions (e.g., retention or removal of the psoas muscle, strengthening the psoas if retained) will affect the patient’s post-surgery ambulatory function.

One option for predicting the complex interaction between custom pelvic prosthesis design, internal hemipelvectomy surgical decisions, post-surgery muscle and hip contact forces, and post-surgery ambulatory function is to use patient-specific neuromusculoskeletal models. In recent years, such models have been used to predict various gait impairments due to stroke (Meyer et al., 2016; Sauder et al., 2019), weakened muscles (Falisse et al., 2019), cerebral palsy (Falisse et al., 2020), and knee osteoarthritis (Fregly et al., 2007). Fregly et al. (2007) predicted a personalized gait modification that minimized an external indicator of medial knee contact force for an individual with medial knee osteoarthritis. The study predicted that subtle gait modifications could reduce the external indicator by an amount comparable to high tibial osteotomy surgery. For an individual post-stroke, Meyer et al. (2016) predicted how the subject would walk at his fastest comfortable speed in excellent agreement with experimental measurements. Sauder et al. (2019) predicted a personalized fast functional electrical stimulation (FastFES) training prescription for an individual post-stroke to improve propulsive force symmetry. The study predicted that optimal muscle selection and stimulation timing could produce a 23% improvement in propulsive force symmetry compared to the standard FastFES prescription. Falisse et al. (2019) predicted various clinical gait deficiencies caused by muscle strength deficits and a lower limb prosthesis that reproduced experimentally observed walking mechanics and energetics. Falisse et al. (2020) also predicted the walking function of a child with cerebral palsy. The study found that the crouch gait pattern observed experimentally was primarily due to altered muscle-tendon properties rather than spasticity or reduced complexity in neuromuscular control. Despite growing predictive simulation capabilities, only a few studies have used them to optimize surgical or rehabilitation treatment decisions (Fregly et al., 2007; Falisse et al., 2019; Pitto et al., 2019; Sauder et al., 2019), and none have explored computational treatment design for individuals receiving internal hemipelvectomy surgery with custom prosthesis reconstruction.

As a first step toward developing such capabilities for internal hemipelvectomy surgery, this study used predictive walking simulations to investigate the effect of post-surgery psoas strength on walking function when custom prosthesis reconstruction is chosen. The simulated surgery mimicked the most common surgical decisions made at MD Anderson Cancer Center in Houston, Texas, under the assumption that the custom prosthesis with total hip replacement was designed to recapitulate the missing pelvic bony anatomy. The computer simulations used a personalized neuromusculoskeletal model of a healthy subject to which the most common surgical decisions were applied. These decisions dictated which lower back and hip muscles were retained and which were removed from the model. To investigate the impact of the operated side psoas muscle on post-surgery walking function, we changed the strength of the psoas from 0% (removed) to 50% (weakened), 100% (pre-surgery), and 150% (strengthened) of its pre-surgery strength. We quantified the impact of the psoas on walking function by calculating stride length, stride time, stance width, spatial symmetry, temporal symmetry, cost of transport, and metabolic cost, along with gait kinematics and kinetics, for all post-surgery walking predictions. The predictive walking simulations provide insight into the potential importance of retaining the psoas muscle for improving post-surgery walking function.

## Methods

### Experimental data collection

Experimental walking data collected previously from a healthy male subject (age 47 years, height 1.7 m, mass 66.7 kg) were used for this study. Data collection was approved by the University of Florida Institutional Review Board, and the subject gave informed consent. Full body marker-based motion capture, ground reaction, and surface and fine-wire electromyographic (EMG) (Table 1, 26 channels from the right leg only) data were collected simultaneously at different walking speeds using a split-belt instrumented treadmill with belts tied. Walking speeds ranged from 1.0 m/s (slower than self-selected) to 1.4 m/s (self-selected) in increments of 0.1 m/s. A highly symmetric representative walking cycle (defined as heel strike to heel strike of the right foot) collected at self-selected speed was chosen for subsequent analysis. Additional experimental data were collected for static standing and isolated lower body joint motion trials. The static standing trial was collected for model scaling purposes. Isolated joint motion trials were collected for each knee and ankle to personalize the model’s lower body joint axes. All functional axes for the joint of interest were exercised during each isolated joint motion trial (Reinbolt et al., 2005, 2008). Pre-existing CT scan data from the subject’s pelvis were also obtained to personalize the pelvis geometry and both hip joints of the scaled generic musculoskeletal model.

**TABLE 1:** Right leg muscles from which surface or fine-wire (*) EMG data were collected.


*Adductor longus
Adductor magnus
Gluteus maximus lateralis
Gluteus maximus medius
Gluteus medius
*Sartorius
Semimembranosus
Semitendinosus
Biceps femoris long head
Rectus femoris
Tensor fascia latae
Gracilis
*Biceps femoris short head
Vastus lateralis
Vastus medialis
*Vastus intermedius
Gastrocnemius medialis
Gastrocnemius lateralis
Tibialis anterior
*Tibialis posterior
*Extensor hallucis longus
*Flexor hallucis longus
Peroneus longus
Soleus
*Extensor digitorum longus
*Flexor digitorum longus

Experimental movement data were filtered using published methods. Ground reaction and marker motion data were low-pass filtered using a fourth-order zero-phase lag Butterworth filter with a cut-off frequency of 7/*t*_*f*_ Hz (Hug, 2011), where *t*_*f*_ is the period of the gait cycle being processed (Meyer et al., 2017). On average, this variable cut-off frequency caused data collected at a normal walking speed to be filtered at approximately 6 Hz. EMG data were high-pass filtered (40 Hz), demeaned, rectified, and low-pass filtered using a fourth-order zero-phase lag Butterworth filter with a cut-off frequency of 3.5/*t*_*f*_ Hz (Meyer et al., 2016). EMG amplitude for each muscle was normalized to the maximum value over all trials. All data were resampled to 101 time points per gait cycle (Meyer et al., 2017).

### Generic model creation

**FIGURE 2:**
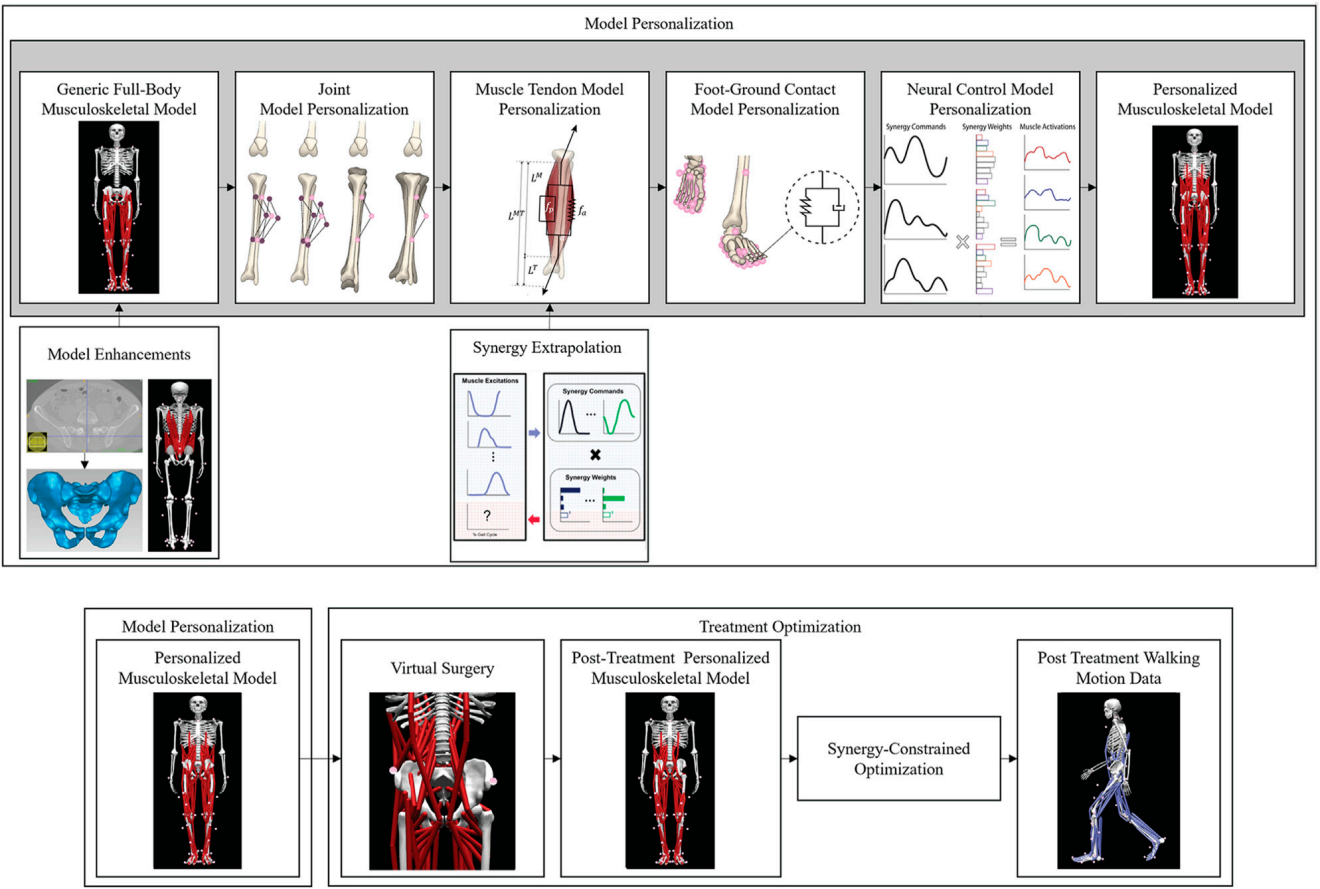
Overview of the computational processes used for model personalization and treatment optimization. Top row: Model personalization process. Bottom row: Treatment optimization process.

A generic full-body OpenSim musculoskeletal model (Rajagopal et al., 2016) (henceforth called the “base model”) was enhanced with additional hip and lower back muscles so that walking function could be predicted following internal hemipelvectomy surgery (Figure 2). The base model possessed 40 Hill-type muscle-tendon actuators per leg and 37 degrees of freedom (DOFs), including a 3 DOF lower back joint, 3 DOF shoulder joints, 1 DOF elbow joints, 3 DOF hip joints, 1 DOF knee joints, 2 DOF ankle joints, and 1 DOF toes joints. The pronation-supination angle of each forearm and the flexion-extension and inversion-eversion angles of each wrist were locked to reduce the number of DOFs to 31.

The hip and lower back muscles added to the base model were taken from two other published generic OpenSim models (Arnold et al., 2005; Bruno et al., 2015). The first was an earlier full-body model (Arnold et al., 2005) that contributed three small hip muscles (i.e., gemelli, pectineus, and quadratus femoris) to each leg of the base model, thereby allowing it to generate the hip internal-external rotation moments required for walking. Since the kinematic structure and bone geometry of the earlier full-body model were identical to those of the base model, the earlier model did not need to be scaled before the six hip muscles were transferred to the base model. The second generic model was a spine model (Bruno et al., 2015) that contributed 68 muscles to each side of the base model’s lower back joint, allowing it to generate the lower back moments required to keep the trunk upright during walking. These muscles included the rectus abdominis, internal oblique, external oblique, erector spinae, multifidus, and quadratus lumborum. Additionally, the psoas was modeled as a hybrid of both models to obtain a physiologically realistic representation that actuated all three degrees of freedom at the hip and lumbar joints. The origin locations and multi-headed psoas muscle from (Bruno et al., 2015) were combined with the wrapping objects and second to fourth insertion locations from (Arnold et al., 2005). Since the bone geometry of the spine model did not match that of the base model, bony landmarks on the pelvis, spine, and ribcage of the spine model and base model were identified in NMS Builder (Valente et al., 2017) and used to determine the affine transformation that mapped the muscle attachments in the spine model onto the base model. The affine transformation and virtual palpation of lower back muscle origins and insertions were performed in accordance with a codified workflow (Modenese et al., 2018). After transfer, muscle attachment locations were adjusted slightly such that the origin and insertion of each muscle head were on the appropriate bone surface. The resulting base model with added hip and lower back muscles possessed 246 Hill-type muscle-tendon actuators.

To simplify the computational prediction of post-surgery walking motions, we performed a model reduction process to minimize the number of heads representing each lower back muscle. The goal was to find the minimum number of original heads with modified peak isometric strength values such that the reduced set of heads produced the same moment-generating behavior as the original heads. This goal was achieved by analyzing all possible combinations of retained heads that spanned the original anatomic range and possessed approximately equal spacing between heads. For each combination, an optimization was performed to identify new peak isometric strength values for the retained heads such that the root-mean-square error between new and original lower back moments was minimized. Lower back moments were calculated for original and retained heads by setting muscle activations equal to one and placing the model in 1,000 random poses selected using Latin hypercube sampling, where the upper and lower bounds for lower back joint angles were chosen to be slightly larger than the joint ranges of motion calculated from the subject’s walking data. For each multiple-head muscle, the combination of retained heads with new peak isometric strength values that most closely matched the moments generated by the original heads was chosen for use in the base model. The resulting reduced model (henceforth called the “enhanced base model”) possessed 148 (74 per side) Hill-type muscle-tendon actuators spanning the lower back, hips, knees, and ankles, where 90 muscles (45 per side) actuated the lower extremities and 58 muscles (29 per side) actuated the lower back. Of particular relevance for the present study, the number of heads representing the psoas muscle was reduced from 11 to 3.

### Personalized model calibration

We followed the first three steps of the four-step model personalization process described in (Meyer et al., 2016; Sauder et al., 2019) to develop a subject-specific walking model (Figure 2). The fourth step involving neural control model personalization was modified as described in the next subsection due to the lack of EMG data for the left leg and all lower back muscles. Prior to running our model personalization process, we scaled the enhanced base model to match the dimensions of the experimental subject using the OpenSim Scale Model tool and the subject’s static standing trial data. Next, the pelvis geometry in the scaled model was replaced with subject-specific pelvis geometry constructed by segmenting the subject’s pelvic CT scan data. Finally, muscle attachment points on the new pelvis geometry were minimally adjusted using NMS Builder (Valente et al., 2017) such that each attachment point was moved to a bone surface. The scaled enhanced base model with the subject-specific pelvis geometry was used for the subsequent model personalization process.

The first step in the model personalization process was joint model personalization. This step involved adjusting the functional axes of the ankle and knee joints in both legs such that the motion of surface markers placed on the model reproduced experimentally measured surface marker motions as closely as possible (van den Bogert et al., 1994; Reinbolt et al., 2005). The adjustment process was formulated as a two-level non-linear optimization problem, where the outer level adjusted joint positions and orientations in their respective segment frames, while the inner level performed repeated OpenSim Inverse Kinematics analyses to calculate root-mean-square errors between model and experimental marker coordinates using the current guess for the joint parameters. The Inverse Kinematics analyses used experimental marker motion data from the isolated ankle and knee motion trials to calibrate each functional axis using a sufficiently large joint range of motion. Since subject-specific pelvis geometry was used for this study, the hip joint locations in the pelvis were defined directly from the pelvis geometry rather than through a functional calibration process.

The second step in the model personalization process was muscle-tendon model personalization. This step involved creating an EMG-driven model of the subject’s right leg (the leg for which EMG data were available), where the inputs to the model were processed EMG signals and lower extremity joint positions and velocities, and the outputs were the net joint moments generated at the ankle, knee, and hip. Prior to constructing the EMG-driven model, we created surrogate models of the subject’s muscle-tendon lengths, velocities, and moment arms as a function of lower extremity joint angles and angular velocities (Menegaldo et al., 2004; Sartori et al., 2012; Meyer et al., 2016, 2017). The surrogate models permitted faster and more reliable calculation of these muscle-tendon geometric quantities than possible by repeatedly calling the OpenSim Muscle Analysis tool. To personalize the muscle-tendon force-generating properties of the right leg muscles, we used a modified version of a published EMG-driven model calibration process (Meyer et al., 2017) to adjust two types of model parameters: EMG-to-activation parameters (electromechanical delays, activation dynamics time constants, activation non-linearization shape factors, and EMG scale factors), and rigid tendon Hill-type muscle model parameters (optimal muscle fiber lengths and tendon slack lengths). Parameter adjustment was performed via non-linear optimization such that the net joint moments produced by the model at the ankle, knee, and hip for walking trials from all available speeds reproduced experimental net joint moments found from OpenSim Inverse Dynamics analyses as closely as possible. Muscle peak isometric strength values were estimated from subject height and weight using published regression relationships derived from MR measurements of lower extremity muscle volumes (Handsfield et al., 2014). Since EMG data were not available for two large (iliacus and psoas) and three small (piriformis, gemelli, and quadratus femoris) hip muscles, we used synergy extrapolation (Ao et al., 2020; Ao et al., 2022) to estimate the missing EMG signals for these muscles.

The third step in the model personalization process was foot-ground contact model personalization. This step involved calibrating the stiffness, damping, and frictional properties of non-linear viscoelastic contact elements placed on a grid across the bottom of each foot such that the contact elements reproduced experimentally measured ground reaction forces and moments as closely as possible. For each foot, the contact element grid was divided into a rear foot and toes grid to permit flexion of the toes segment within the foot model. Each contact element generated a normal force using a linear spring with non-linear damping (Hunt and Crossley, 1975) and a shear force using a continuous stick-slip friction model (Jackson et al., 2016; Meyer et al., 2016). Contact model properties were calibrated for the representative walking cycle using GPOPS-II, a direct collocation optimal control solver (Patterson and Rao, 2014). The calibration process used the full-body model and produced a near-periodic simulated walking motion that matched experimental marker motion for both feet, full body joint angles, lower limb and lower back joint moments, and ground reaction forces and moments as closely as possible while simultaneously satisfying full-body skeletal dynamics (Table 2).

**TABLE 2:** Overview of direct collocation optimal control problem formulations used for model personalization and treatment optimization.

Model personalization				
	Cost Function	Constraints	Static Parameters	Controls
Foot-ground contact model calibration	Track experimental foot marker positions, full-body joint angles, ground reactions, and lower body and back joint moments; Minimize joint jerk	Satisfy skeletal dynamics; Enforce joint angle periodicity	Foot-ground contact model parameters	Joint jerk
Neural control model calibration	Track experimental full-body joint angles, ground reactions, lower body and back joint moments, and muscle activations; Minimize joint jerk	Satisfy skeletal dynamics; Match inverse dynamic lower body and back joint moments using synergy controls; Enforce unit magnitude synergy vectors; Enforce ground reaction and joint angle periodicity	Synergy vector weights	Synergy activations; Joint jerk
Neuromusculoskeletal model verification	Minimize joint jerk	Satisfy skeletal dynamics; Match inverse dynamic lower body joint and lumbar joint moments using synergy controls; Enforce ground reaction and joint angle periodicity	None	Synergy activations; Joint jerk
Strengthening or weakening the psoas muscle	Track synergy activations, track upper body joint angles, minimize joint jerk	Satisfy skeletal dynamics; Match inverse dynamic lower body and back joint moments using synergy controls; Enforce ground reaction and joint angle periodicity; Enforce bounds on muscle activations	None	Synergy activations; Joint jerk

### Muscle activation estimation

Due to the lack of EMG data for the left leg and lower back muscles, neural control model personalization required performing two novel tasks to estimate the missing muscle activations. Both tasks utilized experimental data from the representative walking cycle. The first novel task involved estimating the missing left leg muscle activations from the available right leg muscle activations (see Supplementary Material). First, all muscle-tendon model properties for the right leg were transferred to the left leg. Second, periodicity was imposed on the right leg muscle activations output by activation dynamics in the EMG-driven model. Third, the periodic muscle activations were duplicated for two consecutive gait cycles and fitted with a Fourier series. Fourth, the Fourier-fitted right leg muscle activations were time-shifted by half a cycle to develop an initial estimate of the left leg muscle activations. Fifth, non-linear optimization was used to modify the left leg muscle activations as little as possible such that the ankle, knee, and hip moments generated by left leg muscles matched the corresponding inverse dynamic moments as closely as possible. For each left leg muscle, the optimization modified an activation scale factor, time delay, and aperiodicity term to improve the fit of the left leg muscle moments from inverse dynamics.

The second novel task involved estimating the missing lower back muscle activations (except for psoas) while simultaneously imposing a synergy structure on the muscle activations for each side (Shourijeh and Fregly, 2020). Prior to estimating the missing muscle activations, we minimally adjusted the initial values of optimal muscle fiber length and tendon slack length for each lower back muscle such that every muscle operated on the ascending region of its normalized force-length curve with a maximum normalized length close to one. After this adjustment, non-linear optimization was used to estimate the unknown trunk muscle activations, assuming the activations on each side of the body were generated using a synergy structure (see Supplementary Material). The cost function tracked lower back joint inverse dynamic moments, minimized lower back muscle activations squared, and required muscle heads from the same lower back muscle to have similar activation patterns. The time-varying synergy activations were extracted by decomposing the lower extremity muscle activations using non-negative matrix factorization. The design variables were the time-invariant synergy vector weights for the lower back muscles, where each weight was associated with a corresponding extracted synergy activation. Six synergies were required on each side of the body (Ivanenko et al., 2004) to achieve the best match of inverse dynamic joint moments (all root-mean-square errors <2.5 Nm) and leg muscle activations (98% total variability accounted for).

Once right and left side synergies defining the muscle activations for both legs and the lower back were available, the time-varying synergy activations and associated time-invariant synergy vectors were adjusted slightly to produce a dynamically consistent full-body walking motion that closely reproduced all available data. Synergy adjustments for the representative walking cycle were found using GPOPS-II (Patterson and Rao, 2014), where the cost function tracked experimental full-body joint angles, lower limb and lower back joint moments, experimental ground reaction forces and moments, and experimental and estimated muscle activations. Path constraints were added to satisfy skeletal dynamics and ensure that model-predicted joint moments from inverse dynamics matched muscle-predicted joint moments from synergy-constructed muscle activations. A terminal constraint enforced joint angle periodicity (Table 2).

### Pre-surgery walking verification

The predictive capability of the final personalized model was verified by performing a predictive walking optimization that closely reproduced all experimental data and estimated muscle activations for the representative walking cycle with fixed final time. The verification process involved solving two direct collocation optimal control problems using GPOPS-II. The goal of the first problem was to show that the personalized model could closely reproduce a) experimental full-body joint motions, b) experimental lower back and leg muscle moments, c) experimental ground reaction forces and moments, and d) experimental and estimated muscle activations simultaneously. All of these quantities were tracked in the cost function, which also included a joint jerk minimization term due to the use of an inverse formulation for skeletal dynamics (van Den Bogert et al., 2011; Meyer et al., 2016; Sauder et al., 2019; Febrer-Nafría et al., 2020). The six synergy activations on each side were used as the controls, and the six associated synergy vectors per side were assumed to remain unchanged (Supplementary Figures S1, S2). Path constraints enforced a dynamically consistent solution by eliminating residual forces and torques acting on the pelvis and a kinetically consistent solution by ensuring that joint moments generated by the synergy-constructed muscle activations matched those produced by inverse skeletal dynamics. A terminal constraint enforced joint angle and ground reaction force near-periodicity (Table 2).

The goal of the second problem was to show that the personalized model could reproduce the joint motions, joint moments, ground reactions, and muscle activations found by the first problem simply by tracking the synergy activations produced by the first problem with free final time. The cost function also included minimization of joint jerk and the same path and terminal constraints as the first problem (Table 2). Verification was considered to be successful once the second problem could closely predict the results and final time of both the first problem and the experimental walking motion (Supplementary Table S1).

### Post-surgery walking prediction

A free final time direct collocation optimal control problem was formulated to predict post-surgery walking function for different assumptions about post-surgery psoas strength representative of real-life surgical and rehabilitation scenarios (Figure 2). The optimal control problem assumed that a) the modeled subject’s recovery had plateaued, b) pre-surgery synergy activations for each side changed only minimally to produce the predicted post-surgery walking motion (Meyer et al., 2016; Pitto et al., 2018; Sauder et al., 2019), c) post-surgery walking speed matched the pre-surgery self-selected speed of 1.4 m/s, d) the modeled subject received a custom prosthesis that reproduced the original pelvis and hip anatomy, and e) lower back and hip muscles removed from the right (assumed operated) side of the model (Table 3) corresponded to the most common surgical scenario encountered at MD Anderson Cancer Center in Houston, Texas. Notably, this surgical scenario involves resection of all operated side hip abductor muscles. Muscle removal emulated a worst-case clinical scenario in which all resected muscles were either not reattached or reattached but had no remaining biomechanical function. Four different post-surgery walking predictions were generated for assumed ipsilateral post-surgery psoas strengths of 0%, 50%, 100%, and 150% of pre-surgery strength. Zero percent represented surgical removal of the psoas on the operated side, while 50%, 100%, and 150% represented different potential rehabilitation outcomes when the psoas is retained. These strength values were implemented by changing the peak isometric strength of the ipsilateral psoas muscle. Apart from the addition of free final time, removal of the specified muscles, and inclusion of a path constraint bounding all muscle activations to be between 0 and 1, the optimal control problem formulation was identical to that of the final verification problem (Table 2).

**TABLE 3:** Muscles included in the enhanced base OpenSim musculoskeletal model. Muscles listed in bold text were removed from the operated side of the model when generating post-surgery walking predictions.

Adductor brevis	Gracilis
Adductor Longus	**Iliacus**
Adductor Magnus	Pectineus
Adductor Magnus (distal)	Peroneus Brevis
Adductor Magnus (ischial)	Peroneus Longus
Adductor Magnus (middle)	Piriformis
Adductor Magnus (proximal)	Psoas
Biceps Femoris Long Head	**Quadratus Femoris**
Biceps Femoris Short Head	**Rectus Femoris**
Extensor Digitorium Longus	**Sartorius**
Extensor Hallucis Longus	Semimembranosus
Flexor Digitorium Longus	Semitendinosus
Flexor Hallucis Longus	Soleus
Gastrocnemius Lateral Head	**Tensor Fascia Latae**
Gastrocnemius Medial Head	Tibialis Anterior
**Gemelli**	Tibialis Posterior
Gluteus Maximus	Vastus Lateralis
Gluteus Maximus (inferior)	Vastus Medialis
**Gluteus Maximus (middle)**	Vastus Intermedius
**Gluteus Maximus (superior)**	Rectus Abdominus
**Gluteus Medius**	**Internal Oblique**
**Gluteus Medius (anterior)**	**External Oblique**
**Gluteus Medius (middle)**	**Quadratus Lumborum**
**Gluteus Medius (posterior)**	**Multifidus** ^a^
**Gluteus Minimus**	**Erector Spinae** ^a^
**Gluteus Minimus (anterior)**	
**Gluteus Minimus (middle)**	
**Gluteus Minimus (posterior)**	

^a^ A few heads of these muscles remain.

To quantify the impact of post-surgery psoas strength on predicted post-surgery walking function, we compared multiple clinical walking measures predicted by each of the four optimizations. These included the cost of transport, metabolic cost, stride length, stride time, spatial symmetry, temporal symmetry, and stance width. Cost of transport (CoT) (J/kg*m) is defined as the metabolic cost expended to move a unit of body mass a unit of distance. Metabolic cost is defined as the energy consumed by the body during a given activity, in this case, a single walking cycle. Based on a previous study (Arones et al., 2020), we used Bhargava’s model to calculate the metabolic cost (Bhargava et al., 2004). Stride length is defined as the distance between two consecutive heel strikes of the same foot. Stride time is defined as the time between two consecutive heel strikes of the same foot. Spatial symmetry is defined as the ratio between step length on the operated side and stride length,

$$Spatial\;symmetry=\frac{Operated\;Step\;Length}{Stride\;Length}$$

where step length is defined as the distance between heel strike of one foot and heel strike of the opposite foot. A spatial symmetry value of 0.5 represents perfect symmetry. If the operated foot takes a longer step than does the non-operated foot, then the spatial symmetry value will be less than 0.5. Similarly, if the non-operated foot takes a longer step than does the operated foot, then the spatial symmetry value will be greater than 0.5. Temporal symmetry is defined as the ratio between step time on the operated side and stride time,

$$Temporal\;Symmetry=\frac{Operated\;Step\;Time}{Stride\;Time}$$

where step time is defined as the time between heel strike of one foot and heel strike of the opposite foot. A temporal symmetry value of 0.5 represents perfect symmetry. If the step time for the operated foot is longer than that of the non-operated foot, then the temporal symmetry value will be less than 0.5. Similarly, if the step time for the non-operated foot is longer than that of the operated foot, then the temporal symmetry value will be greater than 0.5.

Stance width is defined as the side-to-side distance between the heel on one foot and the heel on the other foot when each foot is flat on the ground.

## Results

**FIGURE 3:**
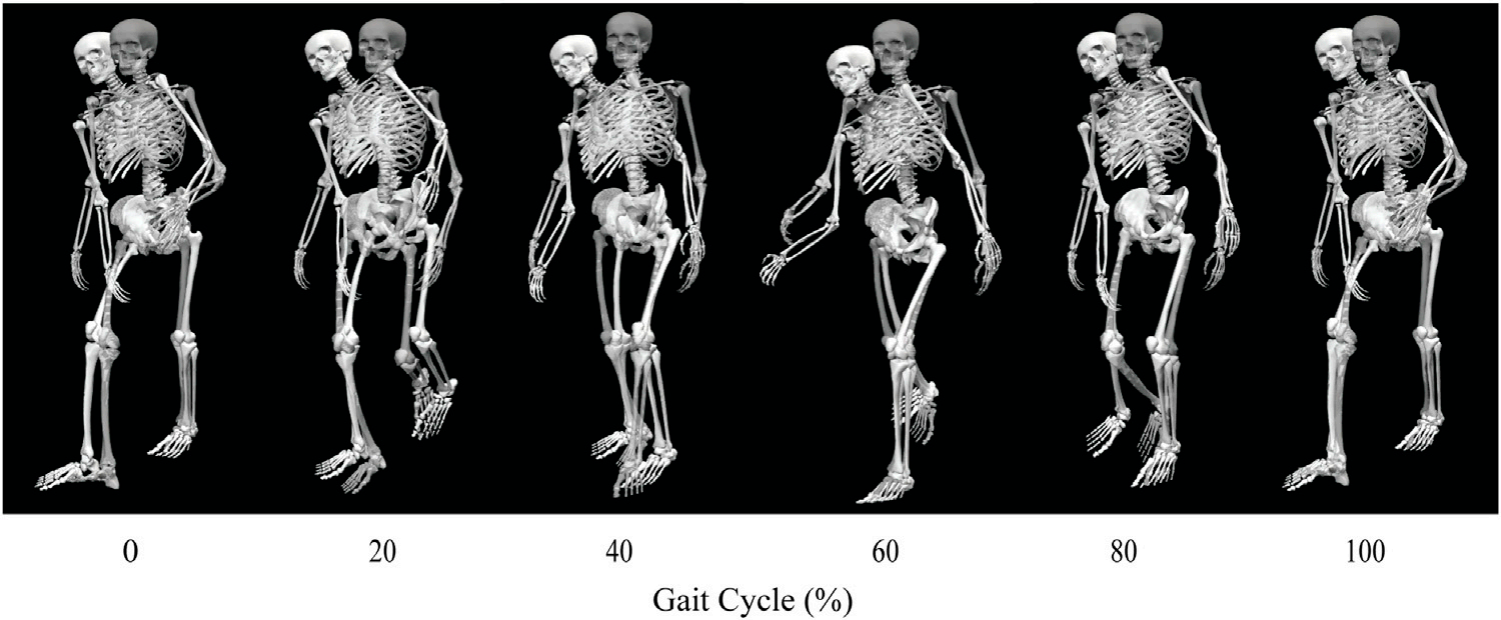
Animation strip comparing the subject’s experimental walking motion (translucent skeleton) to his predicted walking motion when post-surgery operated side psoas strength was 100% of its pre-surgery value (opaque skeleton).

**FIGURE 4:**
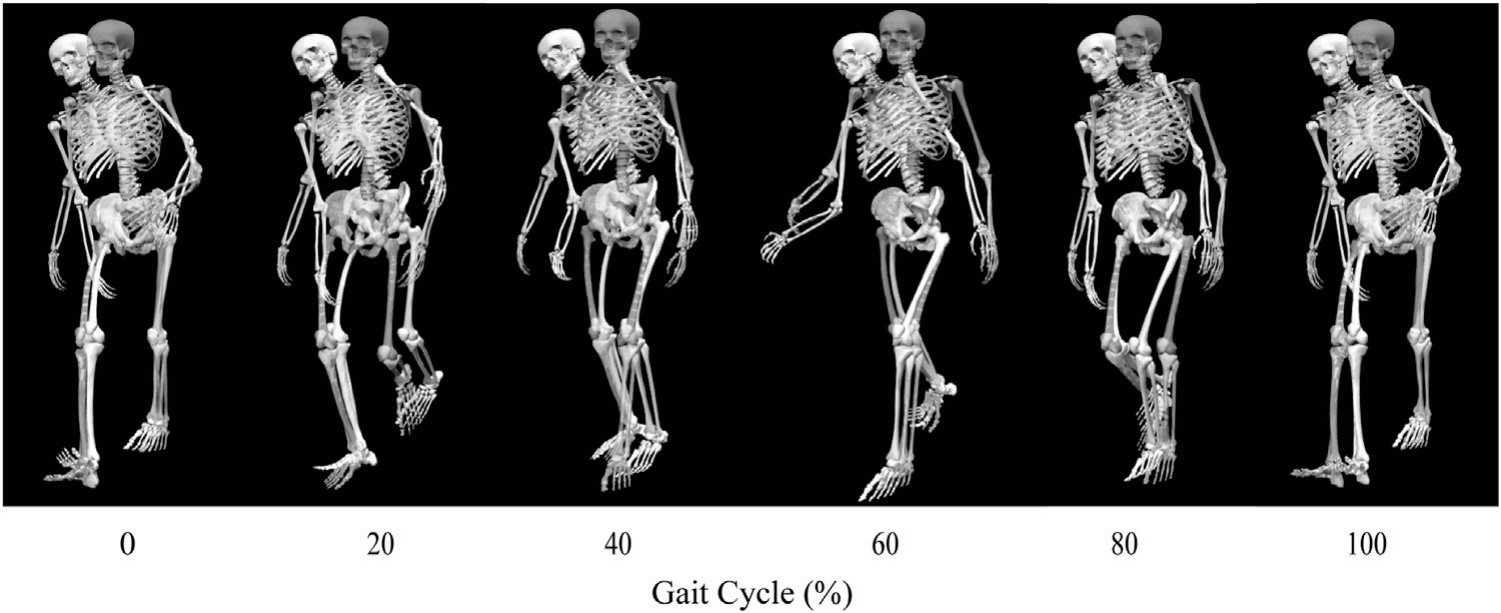
Animation strip comparing the subject’s experimental walking motion (translucent skeleton) to his predicted walking motion when post-surgery operated side psoas strength was 150% of its pre-surgery value (opaque skeleton).

Out of the four post-surgery walking predictions, only the 100% and 150% treatment options successfully converged to a complete gait cycle (heel strike to heel strike of the operated foot). Relative to pre-surgery, the CoT, metabolic cost, spatial and temporal symmetry, and step length decreased while the stance width increased when operated side psoas strength was maintained at its pre-surgery value (100%) (Table 4). However, when the operated side psoas was strengthened above its pre-surgery value (150%), spatial symmetry, stride length, and stance width returned close to pre-surgery values (Table 4). Additionally, cost of transport decreased, metabolic cost increased, and temporal symmetry remained unchanged as post-surgery psoas strength was increased. Both walking predictions possessed a large lateral trunk lean to the operated side (Figures 3, 4), with comparable changes in stride time regardless of post-surgery psoas status.

**TABLE 4:** Comparison of experimental pre-surgery and predicted post-surgery clinical gait measures. Psoas strength is indicated as percent of pre-surgery value

Condition	Psoas strength	CoT $\left(\frac{J}{kg*m}\right)$	Metabolic cost (J)	Spatial symmetry	Temporal symmetry	Stance width (m)	Stride length (m)	Stride time (s)
Pre-Surgery	100%	5.98	620	0.49	0.50	0.082	1.55	1.06
Post-Surgery	150%	5.52	571	0.51	0.47	0.071	1.55	1.10
	100%	5.75	548	0.42	0.47	0.23	1.43	1.05

**FIGURE 5:**
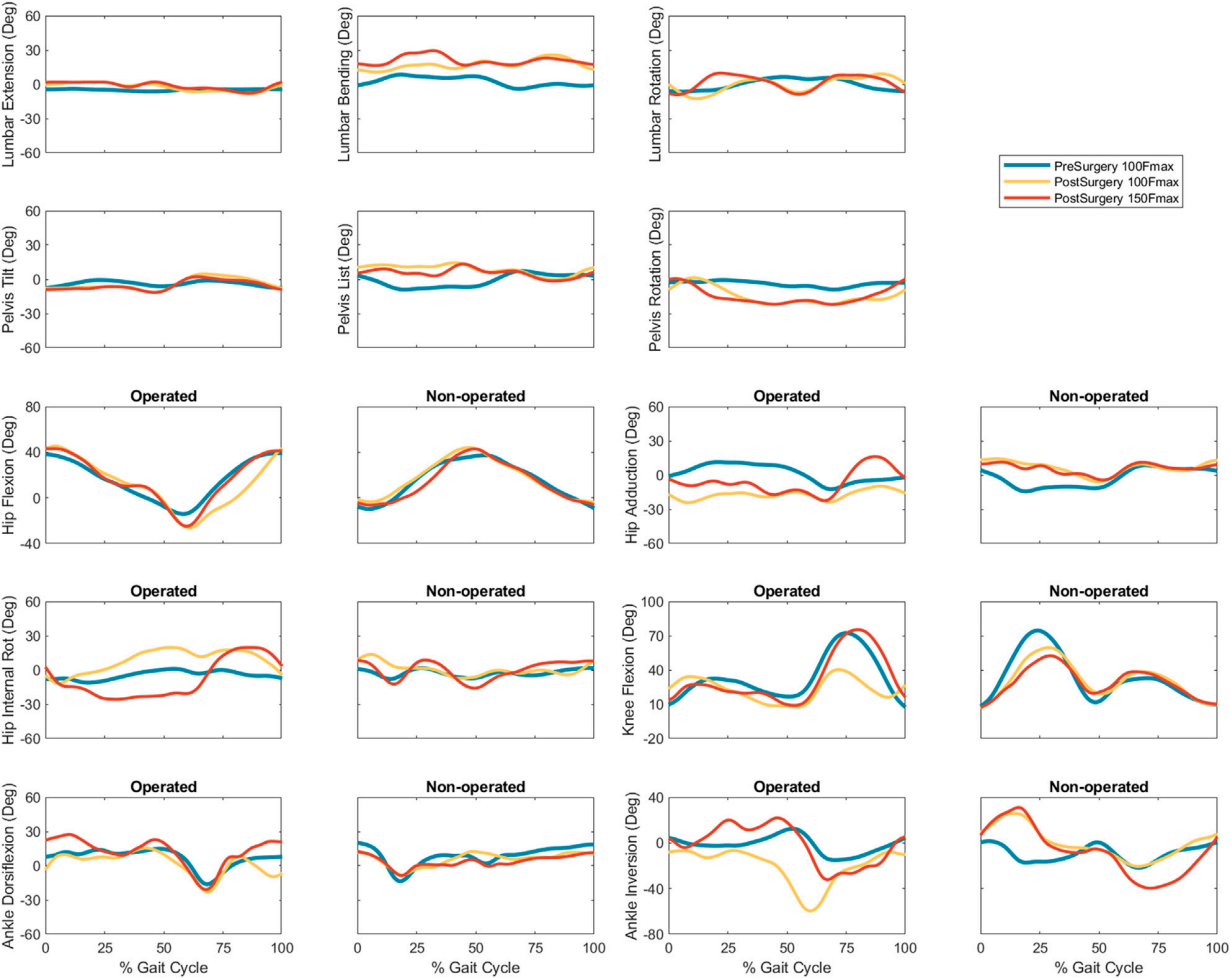
Experimental pre-surgery and predicted post-surgery joint angles for post-surgery psoas strengths of 100%, and 150% of the pre-surgery value. The gait cycle begins with operated leg heel strike.

Predicted joint angles were similar across some joints and substantially different for others between the two successful post-surgery walkng conditions (Table 5; Figure 5). For the operated leg, increased hip extension was observed during toe-off for both post-surgery conditions and during swing phase for the 100% Fmax case. The 100% Fmax case had an increase in hip abduction over the entire gait cycle, while the 150% Fmax had an increase in hip abduction during stance phase and hip adduction during swing phase. For hip rotation, the 100% Fmax case exhibited increased internal rotation over the entire gait cycle, while the 150% Fmax case had increased external rotation during stance phase and increased internal rotation during swing. Knee flexion decreased for the 100% Fmax case during swing phase. At the ankle, the 150% Fmax case had an increase in dorsiflexion during early stance phase and late swing phase. For ankle rotation, the 100% Fmax case exhibited increased eversion over the entire gait cycle, while the 150% Fmax case had increased inversion during stance and increased eversion during swing phase. For the non-operated leg, both post-surgery predictions exhibited increased hip adduction, decreased knee flexion, and increased ankle inversion during swing phase. Additionally, the 150% Fmax case had increased ankle eversion during stance phase. For the lower back, the lumbar bending increased toward the operated side over the entire gait cycle. Although differences in lumbar rotation between the post-surgery conditions were not large in magnitude, the 150% Fmax case displayed pronounced swaying between operated and non-operated sides over the gait cycle.

**TABLE 5:** Root-mean-square (RMS) differences between pre-surgery and predicted post-surgery joint angles. Percentage of Fmax indicates the percent of pre-surgery psoas peak isometric strength used to generate the post-surgery walking prediction.

	RMS difference (deg)	
Joint angle	150% Fmax	100% Fmax
Pelvis Tilt	3.99	4.48
Pelvis List	10.44	13.27
Pelvis Rotation	12.56	12.12
Operated Hip Flexion	5.59	12.54
Operated Hip Adduction	16.09	20.72
Operated Hip Rotation	17.03	14.36
Operated Knee Flexion	9.04	18.13
Operated Ankle Dorsiflexion	8.73	6.79
Operated Ankle Inversion	12.01	25.59
Non-operated Hip Flexion	4.71	4.19
Non-operated Hip Adduction	10.64	12.18
Non-operated Hip Rotation	6.15	6.03
Non-operated Knee Flexion	11.72	8.66
Non-operated Ankle Dorsiflexion	6.24	5.80
Non-operated Ankle Inversion	20.41	15.97
Lumbar Extension	4.78	4.37
Lumbar Bending	18.72	16.02
Lumbar Rotation	8.09	7.15

**FIGURE 6:**
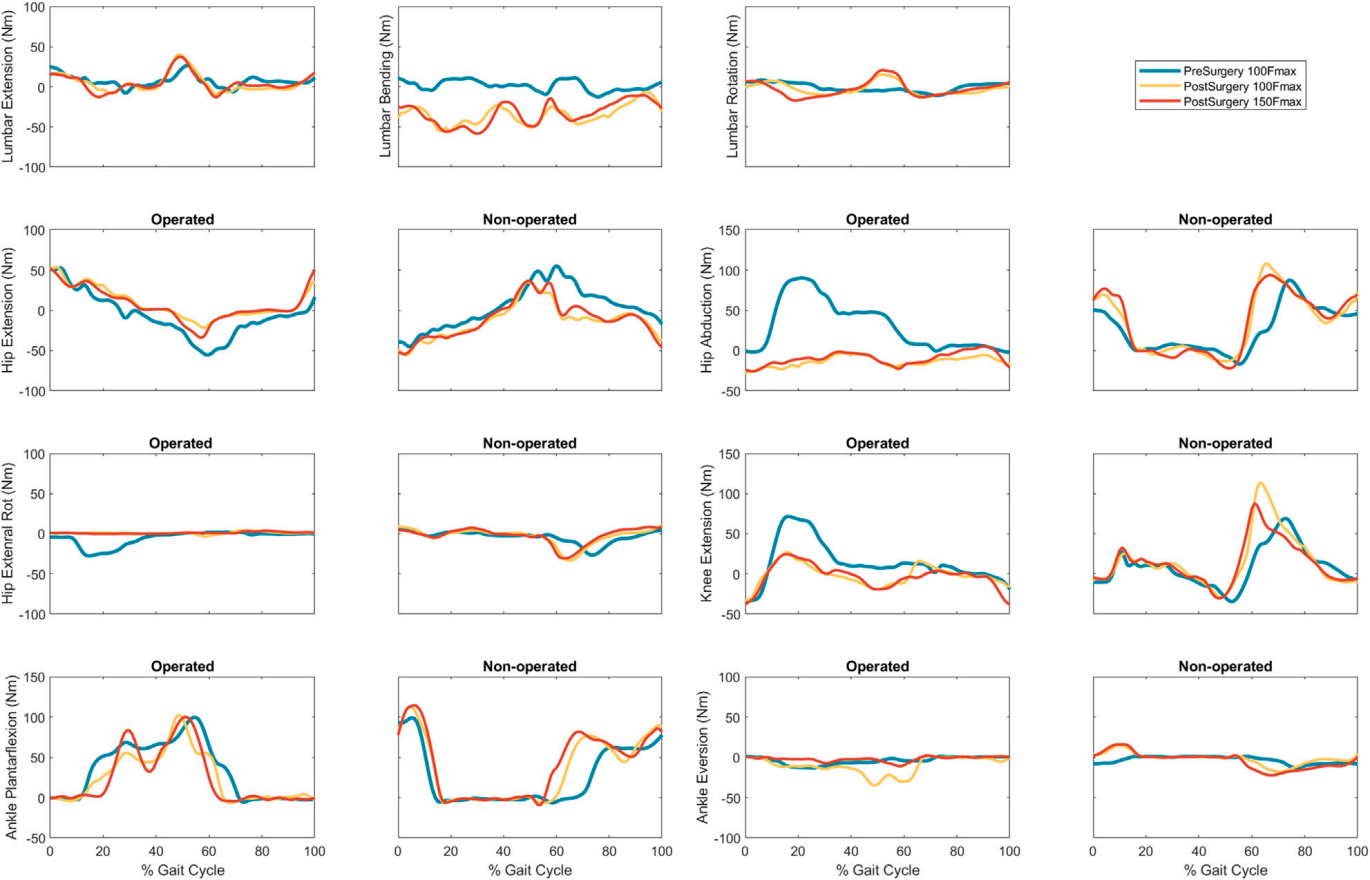
Experimental pre-surgery and predicted post-surgery joint moments for post-surgery psoas strengths of 100%, and 150% of the pre-surgery value. The gait cycle begins with operated leg heel strike.

Predicted lower body joint moments had several large differences compared to pre-surgery walking (Table 6; Figure 6). For the operated leg, the most notable differences were at the hip, where hip abduction and internal rotation moments were close to zero throughout the gait cycle. The hip extension moment followed a similar pattern as pre-surgery but with a decreased hip flexion moment. In addition, peak knee extension moments decreased during stance phase. For the non-operated leg, both post-surgery predictions produced hip flexion, adduction, and internal rotations that followed similar trajectories to those observed experimentally. The largest differences were an increase in hip extension moment during swing, an increase in hip abduction moment during toe-off and early stance, and a time shift in peak hip internal moment. Similarly, the peak knee extension moment time-shifted for both successful post-surgery conditons, but increased in magnitude for the 100% Fmax case. The ankle plantarflexion moment increased during late stance and early swing. Lastly, the lower back bending moment increased toward the non-operated side throughout the gait cycle.

**TABLE 6:** Root-mean-square (RMS) differences between pre-surgery and predicted post-surgery joint moments. Percentage of Fmax indicates the percent of pre-surgery psoas peak isometric strength used to generate the post-surgery walking prediction.

	RMS difference (Nm)		
Joint moment	150% Fmax	100% Fmax	
Operated Hip Flexion	18.07	19.07	
Operated Hip Adduction	19.43	21.70	
Operated Hip Rotation	51.66	54.54	
Operated Knee Flexion	24.91	24.29	
Operated Ankle Dorsiflexion	10.56	11.09	
Operated Ankle Inversion	9.13	7.91	
Non-operated Hip Flexion	23.30	24.05	
Non-operated Hip Adduction	19.59	21.81	
Non-operated Hip Rotation	20.20	16.79	
Non-operated Knee Flexion	28.51	22.61	
Non-operated Ankle Dorsiflexion	4.78	11.16	
Non-operated Ankle Inversion	9.53	7.49	
Lumbar Extension	8.76	8.49	
Lumbar Bending	39.04	39.01	
Lumbar Rotation	10.95	7.89	

In general, the muscle activations produced by the 100% Fmax and 150% Fmax post-surgery walking predictions shared similar characteristics to pre-surgery activations (Supplementary Figures S3–S6). Predicted muscle activations for the operated leg and trunk were comparable to pre-surgery activations with small increases in all three psoas branches, gastrocnemius lateralis, soleus, and the remaining multifidus and erector spinae muscles during late stance phase. Similarly, the muscle activations predicted for the non-operated leg and trunk were similar to pre-surgery activations for both post-surgery conditions, with the exception of small activation increases for iliacus, soleus, piriformis, and tensor fasciae latae.

Analogous to the minimal changes observed in muscle activations, the synergy activations for both post-surgery walking predictions exhibited similar behavior (Table 7, Supplementary Figures S7, S8).

**TABLE 7:** Root-mean-square (RMS) differences between pre-surgery and predicted post-surgery synergy activations. Percentage of Fmax indicates the percent of pre-surgery psoas peak isometric strength used to generate the post-surgery walking prediction.

		RMS difference (unitless)	
Side	Synergy no.	150% Fmax	100% Fmax
Non-operated	1	0.057	0.062
	2	0.065	0.057
	3	0.056	0.067
	4	0.063	0.051
	5	0.057	0.061
	6	0.067	0.068
Operated	1	0.048	0.054
	2	0.067	0.068
	3	0.060	0.064
	4	0.068	0.068
	5	0.067	0.066
	6	0.065	0.048

## Discussion

This study used a personalized neuromusculoskeletal model and direct collocation optimal control to predict the impact of ipsilateral psoas muscle strength on post-surgery walking function following internal hemipelvectomy surgery with custom prosthesis reconstruction. The post-surgery walking prediction problem was formulated to emulate the most common surgical scenario encountered at MD Anderson Cancer Center in Houston, Texas, assuming a custom prosthesis design that recapitulated the original pelvis bony anatomy and hip center location. In addition to removing from the model the same muscles removed surgically, we investigated post-surgery psoas strengths ranging from 0% (removed) to 50% (weakened), 100% (maintained), and 150% (strengthened) of pre-surgery value. For each of the four post-surgery walking situations, a range of clinical gait measures were calculated, including stride length, stride time, step width, spatial symmetry, temporal symmetry, cost of transport, and metabolic cost. Of the four treatment options, only the 100% and 150% scenarios produced successful post-surgery walking predictions. When the strength of the operated side psoas muscle was maintained at its pre-surgery value, the predicted gait pattern exhibited features observed clinically post-surgery (e.g., increased stance width and lateral trunk lean) to accommodate the lack of hip abductor muscles. Interestingly, when the strength of the operated side psoas was increased above its pre-surgery value, stance width, stride length, and spatial symmetry returned to pre-surgery values and the cost of transport decreased. Overall, our post-surgery walking predictions suggest that retention and strengthening of the psoas muscle on the operated side may have a clinically important impact on post-surgery walking ability, at least for the most common surgical scenario involving complete resection of all hip abductor muscles. Other common surgical scenarios, including the second most common scenario at MD Anderson Cancer Center, retain all of the hip abductor muscles, and the influence of the psoas muscle on post-surgery walking function for these scenarios has not yet been investigated.

There are several possible explanations for the inability of the optimal control solver to find a solution for the 0% and 50% Fmax treatment conditions. First, the psoas muscle may play a more critical role in walking than originally anticipated. When post-surgery psoas strength was 100% or 150% of its pre-surgery value, predicted psoas muscle force increased (Supplementary Figures S9) during late stance phase and early swing phase, resulting in a hip flexion moment that was nearly as large as before surgery and a corresponding hip extension angle that was slightly increased. The model was therefore able to use the operated side psoas muscle to swing the operated leg forward to achieve a post-surgery walking pattern that was similar to pre-surgery. Increasing psoas muscle force appears to be metabolically efficient as the cost of transport decreased when post-surgery operated side psoas strength was increased. In contrast, the model lost the ability to complete a full gait cycle when the operated side psoas muscle was either removed or weakened to 50% of its pre-surgery strength, supporting the conclusion that retention of the operated side psoas muscle may be important for maintaining post-surgery walking function. Second, the synergy structure used to predict post-surgery muscle activations may have imposed unrealistic limitations on psoas muscle recruitment. It is possible that following surgery, the synergy vectors coupling muscle activations would be changed such that the psoas could be recruited more heavily without increasing the recruitment of other muscles. Third, hip and lower back muscles taken down during surgery are often reattached to neighboring soft tissue and may still contribute somewhat to walking function. However, reattached muscles may or may not be enervated, and their new attachments may or may not provide mechanical benefits, making it difficult to assess their contribution to post-surgery walking function. A future study should investigate how assumptions about the remaining function of reattached muscles impact post-surgery walking predictions.

Overall, our post-surgery walking predictions exhibited similar gait characteristics to those observed clinically. Compared to pre-surgery, the post-surgery gait patterns exhibited increased stance width through a more lateral foot placement on the operated side, and increased trunk lean toward the operated side. These changes redirected the line of action of the operated side ground reaction force vector through the hip center and up to the center of mass of the trunk, thereby eliminating any ground reaction force moment arm about the hip center. These biomechanical changes allowed the model to accommodate the removal of all hip abductor muscles by eliminating the need for any muscle-generated hip abduction moment.

Though no published experimental studies have reported changes in gait kinematics following internal hemipelvectomy with reconstruction, our predicted increases in stance width and lateral trunk lean were consistent with and larger than, respectively, clinical observations made at MD Anderson Cancer Center in Houston. Several factors may be contributing to this outcome. First, predicted activations for the trunk muscles may have been too low. The synergy-based optimization used to estimate trunk muscle activations essentially ran static optimization with a synergy structure to constrain the solution, and static optimization is known to underestimate muscle co-contraction. Second, reattached trunk muscles may have a substantial contribution to trunk muscle stability. As noted earlier, muscles taken down during surgery are often reattached to neighboring soft tissue, and these reattachments may be especially effective for trunk muscles. Third, the assumed post-surgery optimal muscle fiber lengths and tendon slack lengths of trunk muscles on both sides of the body may have been inaccurate. Changes in these properties alter where a muscle operates on its normalized force-length curve, and it is possible that the body adapts these properties following surgery to place the trunk muscles into a more favorable operating range. This hypothesis is supported by the observation that trunk muscles on the non-operated side produced little to no active force and large passive force from being stretched, while trunk muscles on the operated side produced minimal active force and no passive force from becoming slack due to trunk lean. Future research should investigate the impact of modifying lower trunk muscle-tendon properties on predicted post-surgery walking function. In addition, modifying the spine model to include an additional joint in the thoracic region would allow the shoulders to remain level, as observed clinically, while still allowing the model to lean toward the operated side.

Although both post-surgery simulations predicted two common gait features observed clinically, some of the predicted joint motions were slightly abnormal for this patient population. When post-surgery psoas strength was maintained, the hip internally rotated and the ankle everted more than normal over the entire gait cycle. Similarly, when post-surgery psoas strength was increased, the non-operated leg exhibited increased ankle inversion during swing phase and eversion during stance phase. These abnormal movement patterns may have helped the model compensate for the large number of removed muscles on the operated side. However, this compensatory mechanism could have also resulted from our assumption that the synergy vectors remained fixed and only the synergy activations were allowed to change post-surgery. Since the synergy structure was formulated to control the trunk and legs together, changing a single synergy activation affected multiple muscles. For example, since synergies 2 and 3 possess the largest psoas synergy vectors weights (Supplementary Figures S1, S2, Supplementary Figures S7, S8), increasing these synergy activations also increased muscle activations for other muscles with sizeable weights in the same synergy vectors, including soleus, gastrocnemius lateralis, gastrocnemius medialis, peroneus longus, and peroneus brevis, all of which contribute to subtalar rotation. Similarly, key hip rotation muscles were also affected, including iliacus and tensor fascia latae. Therefore, the abnormal gait features observed in the predicted post-surgery walking motions may be a product of our synergy-controlled problem formulation.

The neural control assumption that the synergy vectors remain fixed and the corresponding synergy activations change as little as possible to produce a new motion has been tested experimentally in only two previous studies (Meyer et al., 2016; Pitto et al., 2018) involving a different patient population than the one here. Thus, this assumption may not be appropriate for the present clinical application involving orthopedic cancer surgery. This hypothesis is supported by the findings of Falisse et al., 2020 (Falisse et al., 2020), who used gait-related performance criteria (e.g., muscle fatigue, energy expenditure) in their optimization cost function to predict a crouch gait pattern using a model of a child with cerebral palsy. Addressing this important issue will require collecting pre- and post-surgery gait data, including video motion capture, ground reaction, and EMG data, from the same patient. Then a personalized model can be constructed for both the pre- and post-surgery situations, and the extent to which the synergy vectors remain unchanged between the two can be evaluated. If the assumption of unchanged synergy vectors turns out to be invalid, such data sets would permit exploration of alternative neural control hypotheses to identify one that works for this particular clinical problem. Any new neural control hypotheses would need to be validated on pre- and post-surgery data sets collected from multiple patients receiving internal hemipelvectomy surgery.

This study represents our initial foray into predicting walking function following internal hemipelvectomy surgery. We decided to focus this initial study specifically on the psoas muscle since its influence on post-surgery walking function is debated among orthopedic oncologists. Since retention of the psoas muscle requires extra time in the operating room, which increases the risk of complications, investigating its importance to post-surgery walking function is a valuable effort. Though these initial post-surgery walking predictions are imperfect, they are still a significant step forward in using computational modeling and simulation to identify optimal surgical, rehabilitation, and even custom prosthesis design decisions for individuals receiving internal hemipelvectomy surgery with or without reconstruction.

Our study possesses several important limitations related to the personalized neuromusculoskeletal model and computational solution approach. First, operated leg EMG data had to be mirrored to the non-operated leg. The mirroring process implicitly assumed that operated and non-operated leg kinematics and kinetics were similar, which was a reasonable assumption for a normal healthy walking cycle. Second, several missing non-operated leg EMG signals had to be estimated. Though previous studies demonstrated that comparable synergy-based EMG estimation methods could predict one missing EMG signal with excellent reliability (Ao et al., 2020; Ao et al., 2022), extension of this methodology to multiple missing EMG signals has yet not been validated. Third, the generic base OpenSim model had to be constructed by combining two different published OpenSim models, one of which had to be modified further to reduce its complexity. Despite our best efforts at combining the two models, it is possible that some trunk muscle lines of action are less accurate than desired. Fourth, even with considerable reduction in the number of trunk muscles, the computation time required to generate each post-surgery walking prediction was large, ranging from 8 to 11 h. Future research efforts will explore ways to reduce computation time to a matter of minutes rather than hours. Fifth, as is the case with any gradient-based optimization, the potential existed for finding a solution that was a local minimum. To minimize the possibility of entrapment in a local minimum, we ran each post-surgery optimal control problem with different initial guesses and reported the solution with the smallest objective function value (Ackermann and van den Bogert, 2010). Sixth (and probably the biggest limitation), no directly comparable post-surgery walking data were available for validation purposes from either the subject modeled in this study (a healthy volunteer) or previous studies. The closest experimental data are from a case study reported by Wingrave and Jarvis 2018 (Wingrave and Jarvis, 2018), which measured post-surgery walking function of a single patient following internal hemipelvectomy. Unlike in our computational study, the patient in that study was not reconstructed with a custom prosthesis, and no list of resected muscles was included, making comparison with our computational predictions infeasible. As noted earlier, it will be critical for future research efforts to measure walking function both before and after internal hemipelvectomy surgery with custom prosthesis reconstruction so that post-surgery walking predictions generated from pre-surgery walking data and the implemented surgical decisions can be validated.

In conclusion, this study used a patient-specific neuromusculoskeletal model to predict post-surgery walking function following internal hemipelvectomy surgery with custom prosthesis reconstruction for different assumptions about post-surgery psoas muscle strength on the operated side. Simulated post-surgery psoas strengths included 0% (removed), 50% (weakened), 100% (maintained), and 150% (strengthened) of the pre-surgery value. However, only the 100% and 150% treatment options successfully converged to a complete gait cycle. When the strength of the psoas was maintained, key clinical post-surgery gait features were observed, including increased stance width, decreased stride length, and increased lumbar bending toward the operated side. Furthermore, when the psoas was strengthened above its pre-surgery level, stance width and stride length returned to pre-surgery values. These results suggest that retention and strengthening of the psoas muscle on the operated side may be important for maximizing post-surgery walking function, at least for the most common surgical scenario involving resection of operated side hip abductor muscles. Other common surgical scenarios retain all of the hip abductors, and thus the influence of the psoas on post-surgery walking function for these scenarios requires further investigation. Future studies comparing computationally predicted and experimentally measured post-surgery walking function for different surgical scenarios will be needed to evaluate and refine our predictive simulation process. Once validated, this computational approach could ultimately influence surgical, rehabilitation, and implant design decisions to meet the unique clinical needs of individual pelvic sarcoma patients.

## Data Availability

The raw data supporting the conclusions of this article will be made available by the authors, without undue reservation.
