# Supplementary material for: Computational evaluation of psoas muscle influence on walking function following internal hemipelvectomy with reconstruction
Source: Front Bioeng Biotechnol. 2022 Sep 28;10:855870. doi: 10.3389/fbioe.2022.855870 (PMC9559731; doi:10.3389/fbioe.2022.855870)
Supplement: Supplementary file 1 [file DataSheet1.pdf]

Supplementary Material

1 Supplementary Information

1.1 Left Leg Muscle Activation Estimation

$$cost = \frac{1}{nDoF \cdot nPts} \sum_{i=1}^{nDoF} \Delta M_i^2 + \left(\frac{1}{nMusc}\right) \sum_{i=1}^{nMusc} \left(\frac{x_i}{0.15}\right)^2 + \left(\frac{1}{nMusc}\right) \sum_{i=1}^{nMusc} \left(\frac{y_i}{0.05}\right)^2$$

$$+ \left(\frac{1}{nMusc_{paired}}\right) \sum_{i=1}^{nPairs} \left(\frac{x_i - \bar{x}}{0.05}\right)^2 + \left(\frac{1}{nMusc}\right) \sum_{i=1}^{nMusc} \left(\frac{z_i}{1.0}\right)^2$$

where

x is activation deviation scale factor, allowable: 0.15, bounds: [-0.30 , 0,30]
y is time delay on activation, allowable: 0.05 sec, bounds: [-0.10, 0.10] sec
z is activation curve tilt, allowable: 1 deg, bounds: [-4.0, 4.0] deg

1.2 Lower Back Muscle Activation Estimation

Li et al. (2022) developed a method to estimate trunk muscle activations during gait using muscle synergy structure. This method is based on the evidence that muscle synergies are shared between lower extremity muscles and trunk muscles. Non-negative matrix factorization was used to decompose the estimated lower extremity muscle activations to find the time-varying synergy activations that were considered to be shared between both the trunk and the lower extremity muscles on each side of the body. A numerical optimization was then used to find the time-independent synergy vector weights for the trunk muscles. The optimization iteratively searched for synergy vector weights, combined with the synergy activations that could form the trunk muscle activations which minimized the objective function.

$$J = J_M + J_a + J_{a\ band\ dev} + J_{a\ dev} + J_{w\ dev}$$

Expanding each term in the objective function,

$$J_M = \frac{1}{nPt \cdot nDoF} \sum_{i=1}^{nPt} \sum_{j=1}^{nDoF} \left(\frac{M_{est\,i,j} - M_{ID\,i,j}}{Allow_{\Delta M}}\right)^2$$

where, nPt = 101 to represent each percent point during the gait cycle. M_{est} is the lower back joint moment estimated given the trunk muscle activations, the Hill-type muscle model parameters, muscle tendon lengths and moment arms about the lower back degrees of freedom for the trunk muscles. M_{ID} is the inverse dynamics joint moment.

$$J_a = \frac{1}{nPt \cdot nMusc} \sum_{i=1}^{nPt} \sum_{j=1}^{nMusc} \left(\frac{a_{est\,i,j}}{Allow_a}\right)^2$$

where, a_{est} is the estimated muscle trunk activations.

$$J_{a\ band\ dev} = \frac{1}{nPt \cdot nMusc} \sum_{i=1}^{nPt} \sum_{j=1}^{nMusc} \left(\frac{a_{est_{i,j}} - a_{center}}{Allow_{\Delta\ a\ band}} \right)^2$$

where, a_{center} is the target muscle activation level.

$$J_{a\ dev} = \sum_{i=1}^{nGrp} \left[\frac{1}{nPts \cdot nMiGrp} \sum_{j=1}^{nPts} \sum_{k=1}^{nMiGrp} \left(\frac{a_{est\ j,(i,k)} - \bar{a}_{estGrp_{j,i}}}{Allow_{\Delta a}} \right)^2 \right]$$

where, $nGrp$ = 7 for the seven trunk muscle groups in the model: rectus abdominus, external obliques, internal obliques, erector spinae, multifidus, and quadratus lumborum. $nMiGrp$ represents number of muscle heads within each of the trunk muscle groups. $\bar{a}_{estGrp}$ is the mean activation across the muscle heads within the same trunk muscle group.

$$J_{w\ dev} = \sum_{i=1}^{nGrp} \left[\frac{1}{nSyn \cdot nMiGrp} \sum_{j=1}^{nSyn} \sum_{k=1}^{nMiGrp} \left(\frac{w_{est\ j,(i,k)} - \bar{w}_{estGrp_{j,i}}}{Allow_{\Delta w}} \right)^2 \right]$$

where, $nSyn$ = 6 because six muscle synergies were used to estimate trunk muscle activations on each side of the body. w_{est} is the set of synergy vector weights associated with each synergy activation for the trunk muscles, estimated by the optimization. $\bar{w}_{estGrp}$ is the mean of synergy vector weights across the muscle heads within the same trunk muscle group for a given muscle synergy.

Six synergies were chosen unilaterally because subtracting one synergy would reduce the quality of moment tracking. Furthermore, six synergies reconstruct the EMG activations of leg muscles in each side with a VAF > 0.98.The optimization was performed using MATLAB's *fmincon* function.

References

Li, G., Ao, D., Vega, M.M., Shourijeh, M.S., Zandiyeh, P., Chang, S.-H., Lewis, V.O., Dunbar, N.J., Babazadeh-Naseri, A., Baines, A.J., and Fregly, B.J. (2022) Estimation of trunk muscle activations during gait using lower extremity muscle synergies. *Frontiers in Bioengineerinig and Biotechnology* (in review).

Shourijeh, M. S., and Fregly, B. J. (2020). Muscle synergies modify optimization estimates of joint stiffness during walking. *J. Biomech. Eng.* 142. doi:10.1115/1.4044310/955411.

2 Supplementary Figures and Tables

2.1 Supplementary Figures

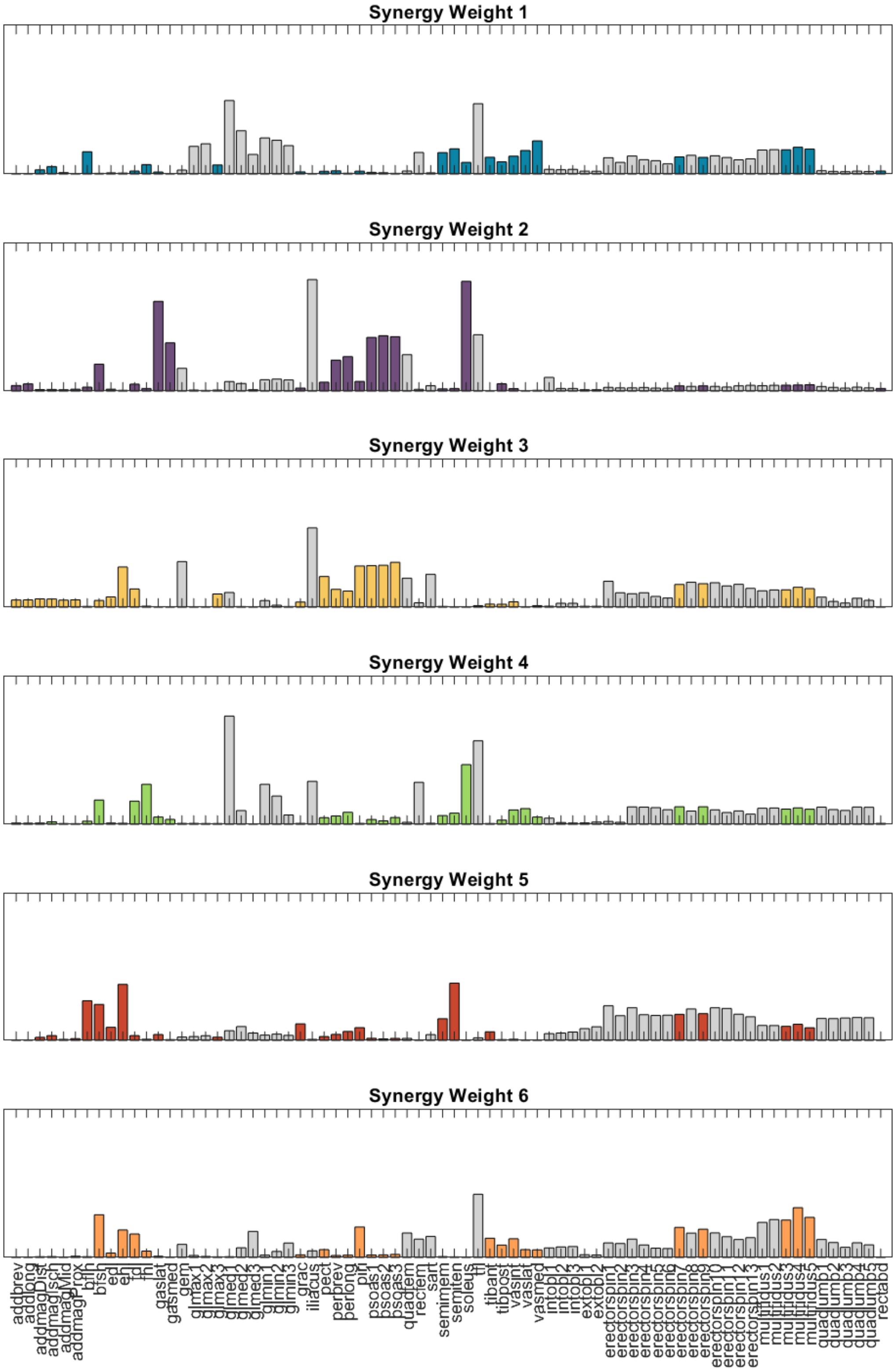

Figure S1. Operated side synergy vectors for pre- and post-surgery conditions. Synergy vector weights shown in gray reflect muscles removed from the model to simulate the post-surgery situation.

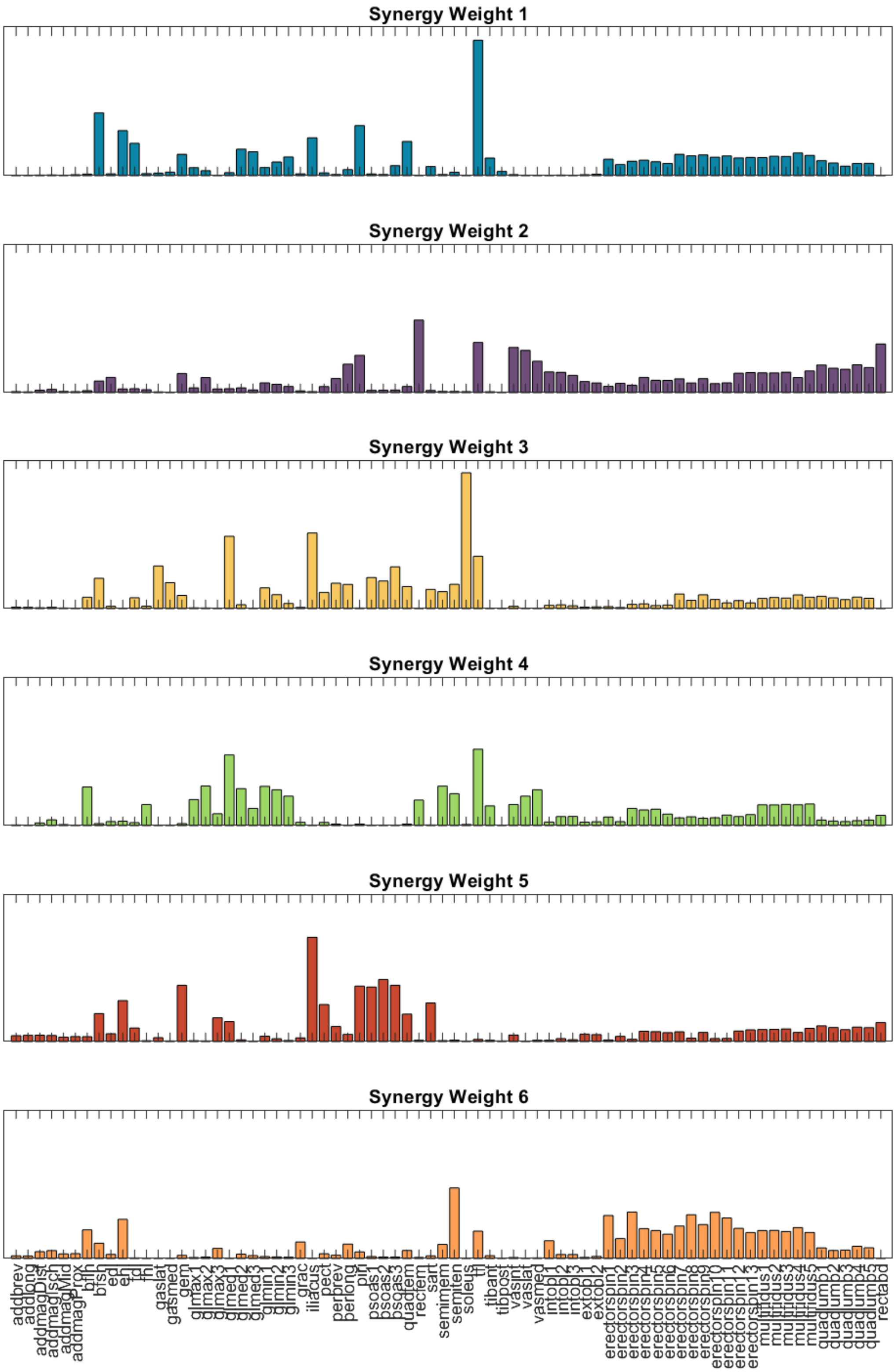

Figure S2. Non-operated side synergy vectors for pre- and post-surgery conditions.

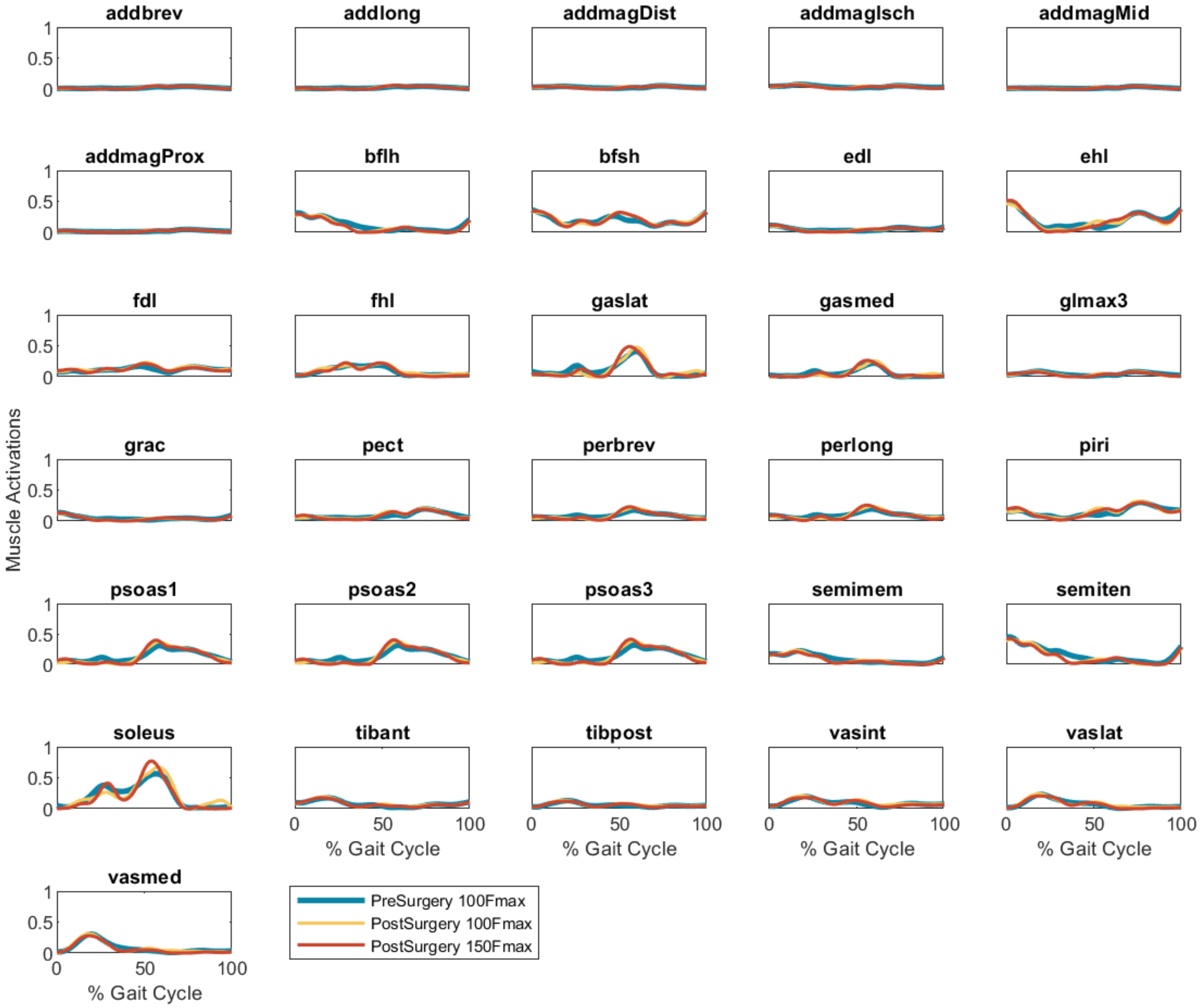

Figure S3. Experimental pre-surgery and predicted post-surgery operated side leg muscle activations for post-surgery psoas strengths of 100%, and 150% of the pre-surgery value.

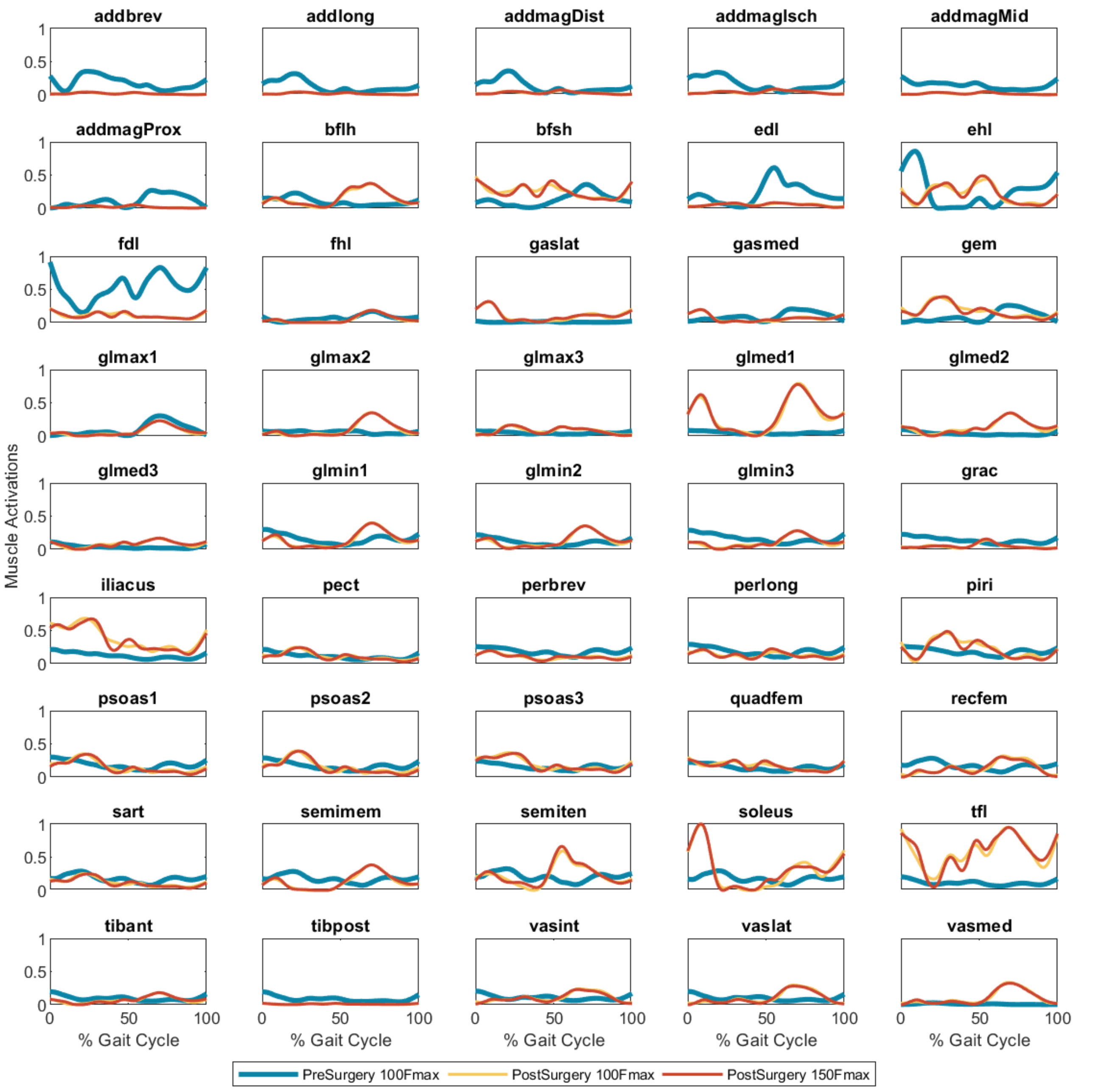

Figure **S4.** Experimental pre-surgery and predicted post-surgery non-operated side leg muscle activations for post-surgery psoas strengths of 100%, and 150% of the pre-surgery value.

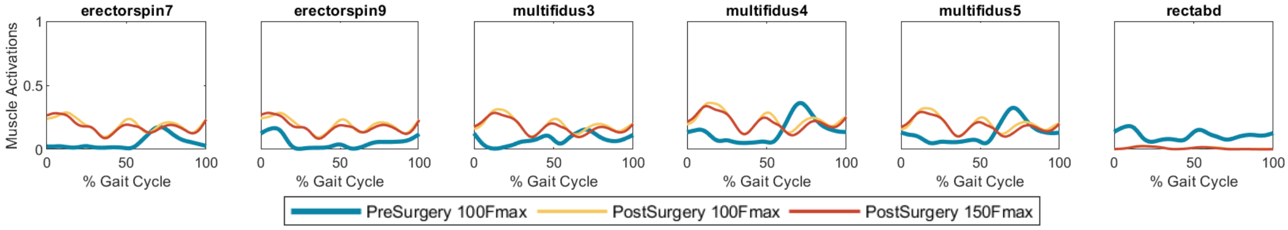

Figure S5. Experimental pre-surgery and predicted post-surgery operated side lower back muscle activations for post-surgery psoas strengths of 100%, and 150% of the pre-surgery value.

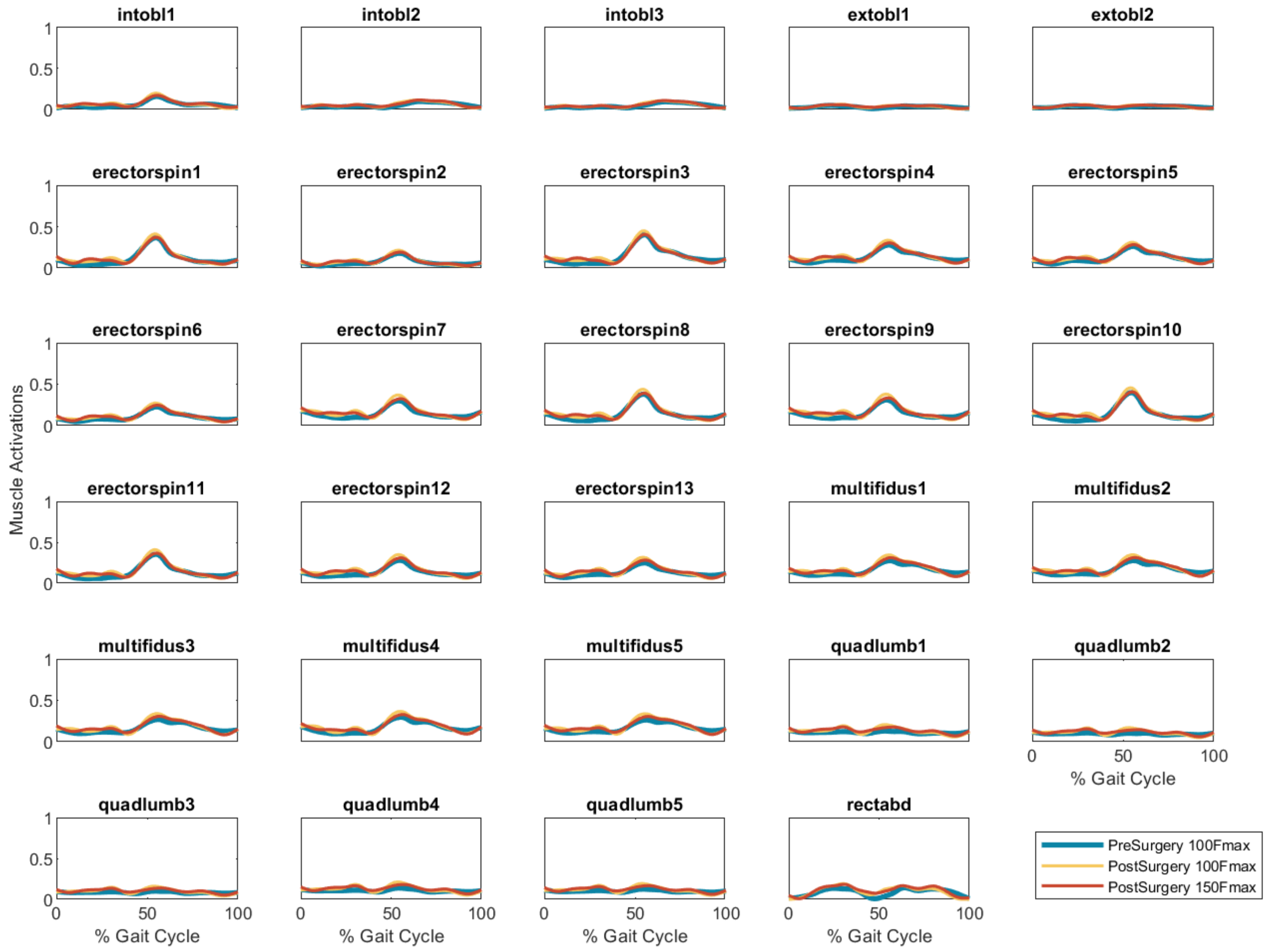

Figure S6. Experimental pre-surgery and predicted post-surgery non-operated side lower back muscle activations for post-surgery psoas strengths of 100%, and 150% of the pre-surgery value.

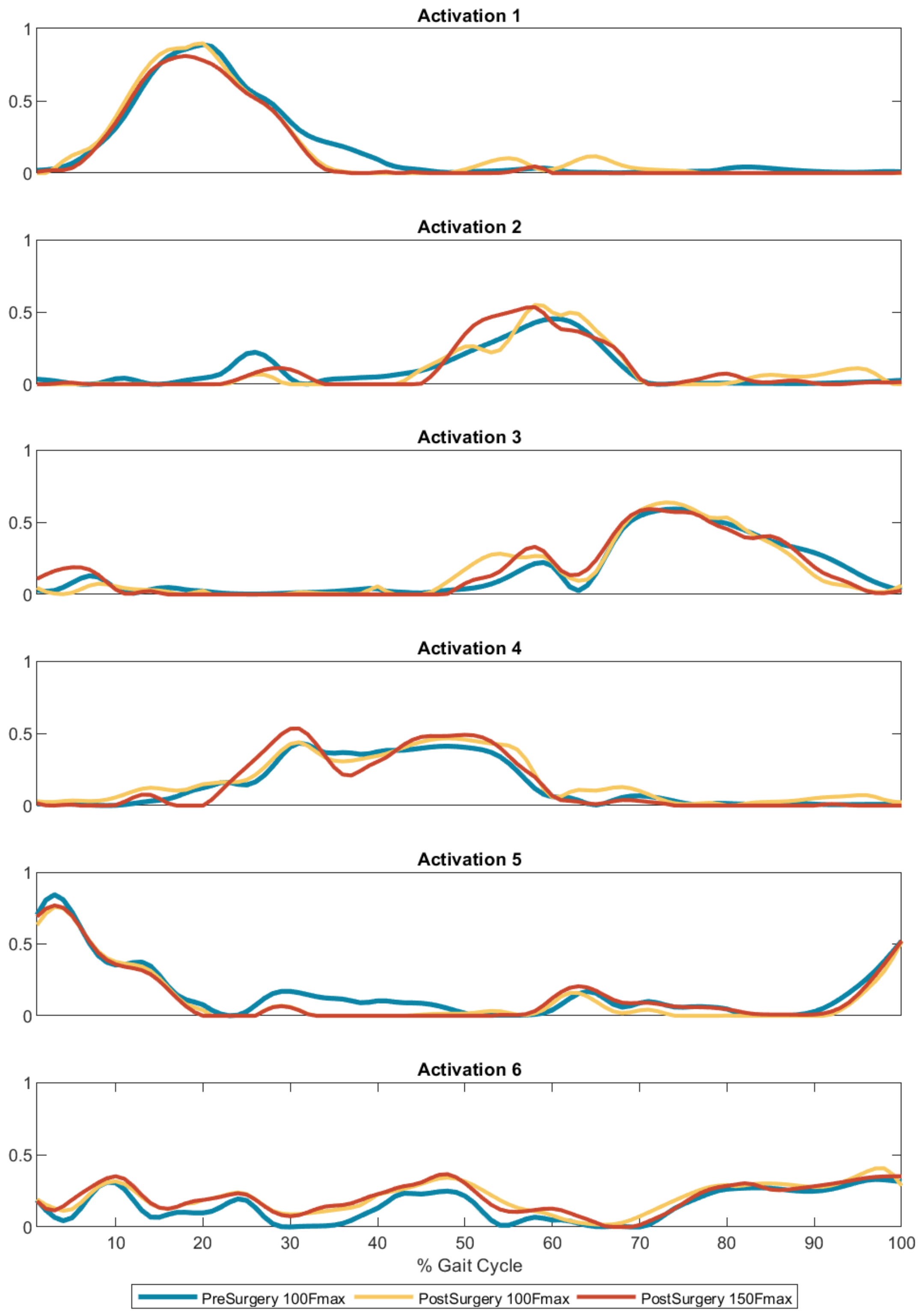

Figure S7. Experimental pre-surgery and predicted post-surgery operated side synergy activations for post-surgery psoas strengths of 100%, and 150% of the pre-surgery value.

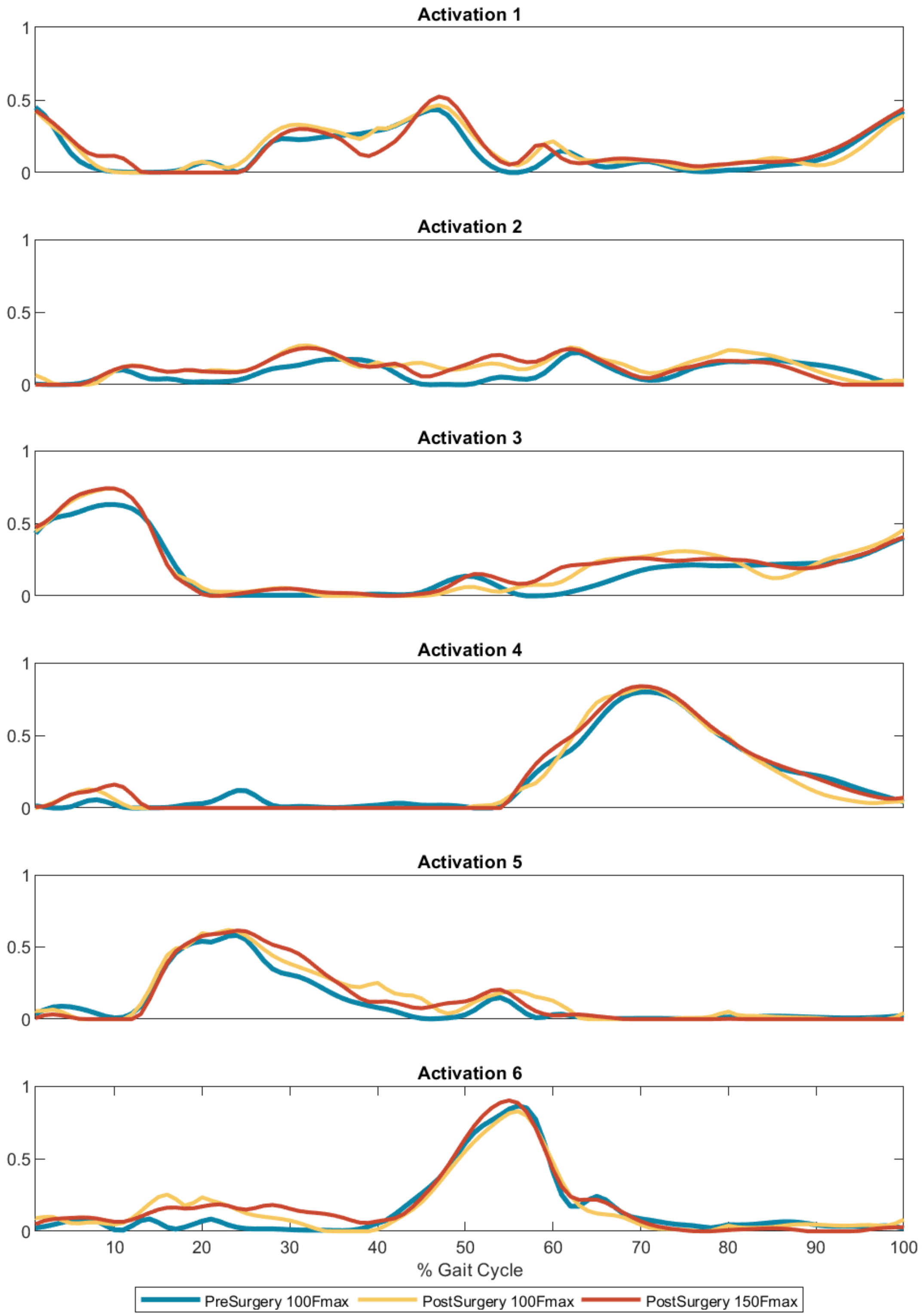

Figure S8. Experimental pre-surgery and predicted post-surgery non-operated side synergy activations for post-surgery psoas strengths of 100%, and 150% of the pre-surgery value.

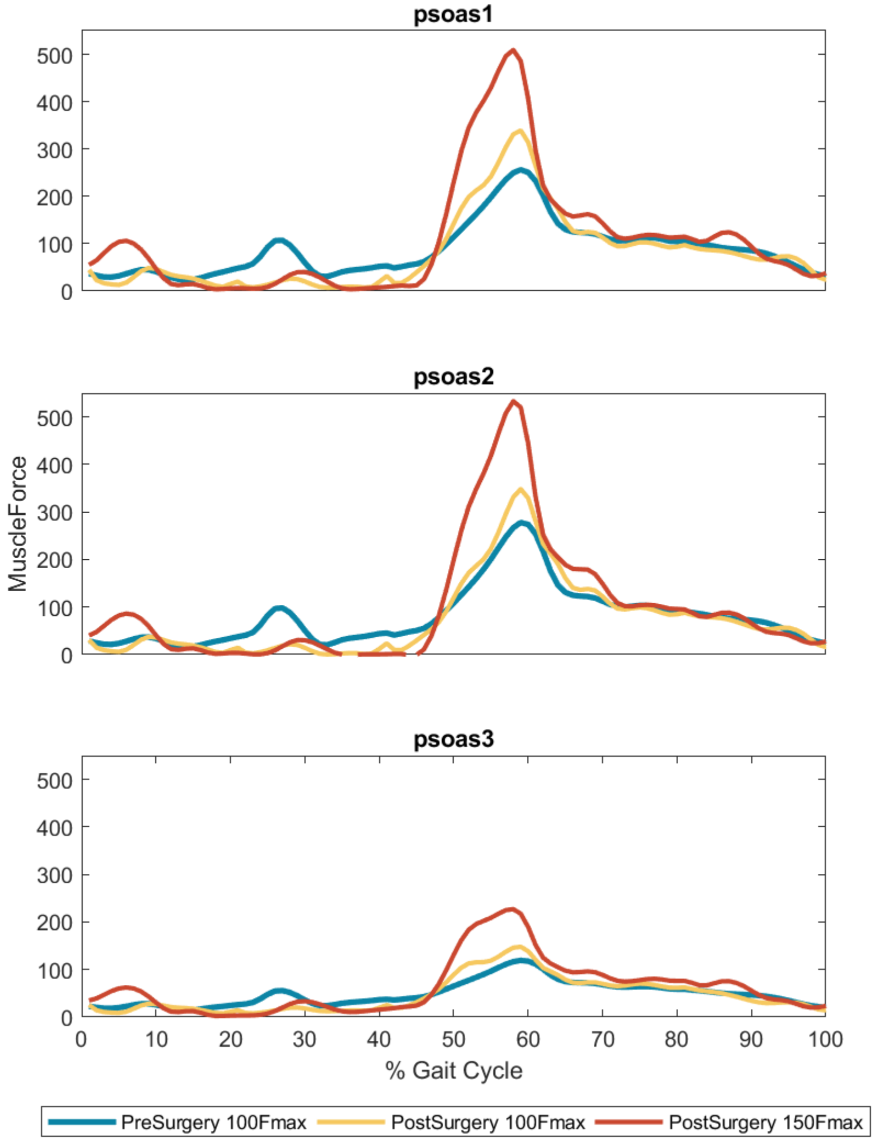

Figure S9. Experimental pre-surgery and predicted post-surgery operated side psoas muscle forces [N] for post-surgery psoas strengths of 100% and 150% of the pre-surgery value. The pre-surgery Fmax values for the three-headed psoas are 657.1, 546.2, 340.3 N.

2.2 Supplementary Tables

Table S1. Average root-mean-square error (RMSE) in joint angles, joint moments, ground reaction forces and moments, and final time between pre-surgery experimental and verification optimization walking patterns.

Quantities	RMSE
Joint Angles (deg)	0.31
Joint Moments (Nm)	3.32
Ground Reaction Forces (N)	9.42
Ground Reaction Moments (Nm)	4.48
Final Time (sec)	0.0043
